# An Inorganic Click Reaction for the Synthesis of Interlocked Molecules

**DOI:** 10.1002/anie.202309211

**Published:** 2023-08-11

**Authors:** Alex Mapp, Jamie T. Wilmore, Paul D. Beer, Jose M. Goicoechea

**Affiliations:** ^1^ Department of Chemistry University of Oxford Chemistry Research Laboratory 12 Mansfield Rd. Oxford OX1 3TA UK; ^2^ Department of Chemistry Indiana University 800 East Kirkwood Ave. Bloomington IN 47405 USA

**Keywords:** Azide, Click Chemistry, Cyaphide, Rotaxanes, Triazaphospholes

## Abstract

We describe the use of the cyaphide‐azide 1,3‐dipolar cycloaddition reaction for the synthesis of a new class of inorganic rotaxanes containing gold(I) triazaphosphole stoppers. Electron‐deficient bis‐azides, which thread perethylated pillar[5]arene in aromatic solvents, readily react with two equivalents of Au(IDipp)(CP) (IDipp=1,3‐bis‐(2,6‐diisopropylphenyl)‐imidazol‐2‐ylidene) to afford interlocked molecules via an inorganic click reaction. These transformations proceed in good yields (ca. 65 %) and in the absence of a catalyst. The resulting organometallic rotaxanes are air‐ and moisture‐stable and can be purified by column chromatography under aerobic conditions. The targeted rotaxanes were characterized by multi‐element nuclear magnetic resonance (NMR) spectroscopy, mass‐spectrometry, and single‐crystal X‐ray diffraction.

## Graphical abstract

A quantitative inorganic click reaction that proceeds in solution in the absence of a catalyst has been used as a stoppering strategy for the synthesis of interlocked molecules in good yields (ca. 65 %). The resulting organometallic rotaxanes are air‐ and moisture‐stable and can be purified by column chromatography under aerobic conditions.

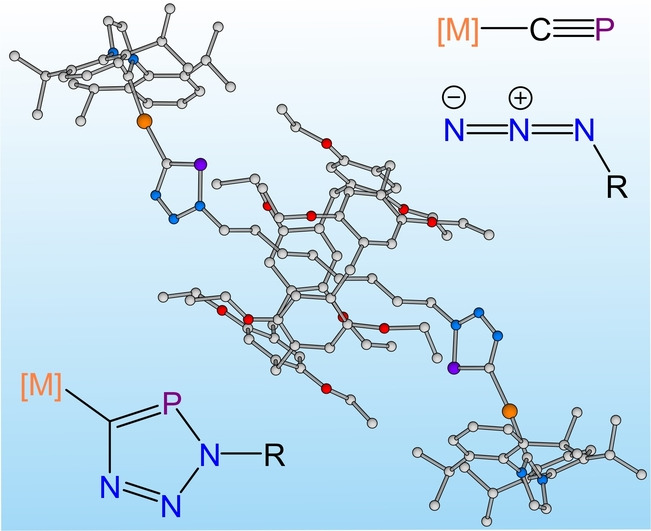

The copper(I)‐catalyzed azide‐alkyne click (CuAAC) reaction has become a widely used tool in the synthesis of mechanically bonded interlocked molecules. It is most commonly employed in active‐metal templation reactions, in which a copper(I) ion plays a dual role: pre‐organizing molecular components and catalyzing interpenetrative covalent bond formation.^[1]^ Alternatively, it can be used solely for the latter purpose in rotaxane stoppering or catenane cyclization reactions, as it is a selective and high yielding reaction, and can be carried out in relatively non‐polar solvents that maximize secondary templating strategies.^[2]^ Numerous other metal ions and metal‐containing compounds have also been employed as templates or catalysts for the synthesis of interlocked molecules, however these are rarely incorporated into the final product.^[3]^

The most common strategy for incorporation of metal complexes into supramolecular molecules is to exploit their Lewis acidity through complexation with basic moieties. The use of this strategy as a capping technique for the synthesis of interlocked molecules was pioneered by Ogino who showed that dative bond formation between a *pseudo*‐rotaxane (threaded by a long chain aliphatic bis‐amine) and cobalt(III) complexes could be used to access rotaxanes, albeit in low yields (ca. 7 %).[^4^, ^5^] Since then, the use of dative bonds has been extensively employed in supramolecular chemistry.[^6^, ^7^, ^8^, ^9^] Strategies that allow for incorporation of organometallic sub‐units into supramolecular systems through formation of non‐dative—and hence intrinsically less labile—covalent bonds are rare and predominantly involve the use of sub‐components with alkynyl functional groups. Gladyz and co‐workers have demonstrated that an active‐metal templation method can be used to couple two platinum(II) tetrayne complexes in the cavity of a macrocycle to access rotaxane‐based molecular wires.[^10^, ^11^] Similarly, Wang has shown that platinum(II) capped rotaxanes (and rotaxane dendrimers) are accessible through metalation of *pseudo*‐rotaxanes threaded by molecules with alkynyl end‐groups.^[12]^ Hybrid organic–inorganic rotaxanes prepared via secondary ammonium hydrogen bonding interactions with polychromium/cobalt and {Ti_7_M^III^} (M=Fe, Ga, Cr, Mn) metal rings have been reported by Leigh and Winpenny.[^13^, ^14^] Pöthig has utilised the excellent host–guest properties of organometallic pillarplex macrocycles to access hybrid organic–inorganic rotaxanes.^[15]^ More recently, John and Szyszko have prepared rotaxanes containing cubic silsequioxane stoppers via the CuAAC reaction in an active‐metal templating route with pre‐functionalized alkynes and azides.^[16]^

The relative rarity of “inorganic rotaxanes” is due to the dearth of reliable methods for generating mechanically interlocked molecules containing inorganic or organometallic sub‐components. This prompted us to ask whether a versatile and high‐yielding synthetic protocol, akin to the CuAAC reaction, could be used to access supramolecular compounds with inorganic functionalities. Phosphaalkynes (R−C≡P), which are valence isoelectronic with alkynes, are known to undergo cycloaddition reactions with organic azides to selectively produce 3*H*‐1,2,3,4‐triazaphospholes, in a manner reminiscent of the CuAAC reaction.[^17^, ^18^, ^19^, ^20^, ^21^, ^22^, ^23^, ^24^, ^25^] Unfortunately, the synthesis and handling of phosphaalkynes is challenging due to their pyrophoric nature.^[26]^ Müller and co‐workers have recently demonstrated that the cyaphide ion (C≡P^−^)^[27]^ can be stabilized in the coordination sphere of platinum, and that the resulting complex subsequently undergoes a cycloaddition reaction with Dipp‐N_3_ (Dipp=diisopropylphenyl).^[28]^ This hints at an interesting potential strategy for the synthesis of interlocked molecules containing organometallic moieties.

We have recently developed a cyaphide‐transfer reagent with Grignard‐like reactivity that allows access to a variety of cyaphido metal complexes.[^29^, ^30^, ^31^] We have also gone on to show that inorganic click reactivity is common to a wide range of metal complexes containing a cyaphide ligand.^[32]^ These cycloaddition reactions proceed rapidly, and in non‐polar solvents, to selectively afford the metallo‐triazaphospholes in high yields. We reasoned that this could offer a general route to incorporating both organometallic moieties and donor phosphorus atoms into interlocked molecules. The synthesis of rotaxanes with phosphorus(III) donors poses a synthetic challenge^[33]^ despite the potential application of such compounds as stimulus‐responsive catalysts.[^34^, ^35^] Herein, we use the cyaphide‐azide 1,3‐dipolar cycloaddition reaction in a capping strategy to synthesize two inorganic rotaxanes containing metallo‐triazaphosphole stoppers.

For a simple [2]rotaxane target we chose perethylated pillar[5]arene (**P5A**) as a macrocyclic host due to its facile synthesis, good solubility in aromatic solvents, and excellent host–guest properties when combined with electron‐deficient guests.[^36^, ^37^, ^38^] The simple long chain bis‐azide, 1,8‐bis‐azido‐octane (**1a**), threads **P5A** with a weak binding affinity (K=122 M^−1^) in C_6_D_6_ (Scheme 1). In order to increase the formation of the pseudo‐rotaxane (**2a**), five equivalents of **P5A** were used to shift the equilibrium towards **2a**. Subsequently, reaction with two equivalents of Au(IDipp)(CP) was used to form the [2]rotaxane (**3a**), featuring gold‐triazaphosphole stoppers possessing a bulky and strongly bonded IDipp ligand which prevents dethreading (Scheme 1).^[39]^

**Scheme 1:**
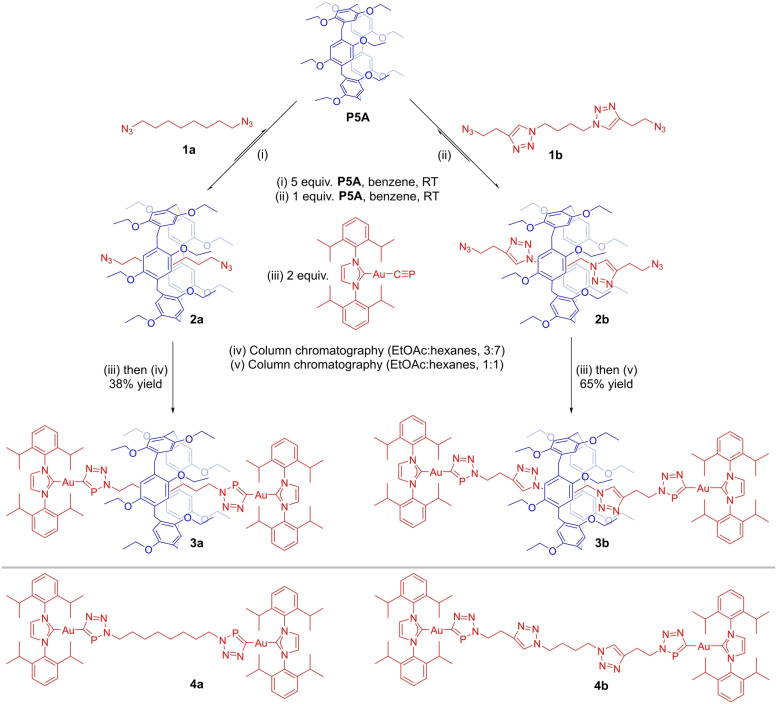
Top: Synthesis of [2]rotaxanes **3a** and **3b**; Bottom: Non‐interlocked axles **4a** and **4b**.

The reaction was monitored by ^31^P{^1^H} NMR spectroscopy, with quantitative consumption of Au(IDipp)(CP) after a few hours at room temperature. The formation of **3a** was evidenced by the emergence of a new peak in the ^31^P{^1^H} NMR spectrum at 198.6 ppm, alongside the non‐interlocked axle **4a** which was observed at 199.9 ppm (approx. 10 : 1 ratio **3a**:**4a**). **4a** could be independently synthesized by the reaction of 1,8‐bis‐azido‐octane **1a** with two equivalents of Au(IDipp)(CP) in the absence of **P5A** (see Supporting Information for details). The ^31^P NMR chemical shifts for **3a** and **4a** are comparable to those of other triazaphospholes.^[32]^ Crystals of **3a** suitable for single‐crystal X‐ray diffraction were obtained from the reaction mixture upon standing, and confirmed the formation of the [2]rotaxane (Figure 1). Remarkably, **3a** required no special handling and could be isolated in moderate yields (38 %) by filtration followed by column chromatography. The air‐ and moisture‐tolerance of this species is greater than that of the non‐interlocked thread **4a**, presumably due to the kinetic stabilization of the reactive triazaphosphole moiety by the pillar[5]arene macrocycle.

**Figure 1:**
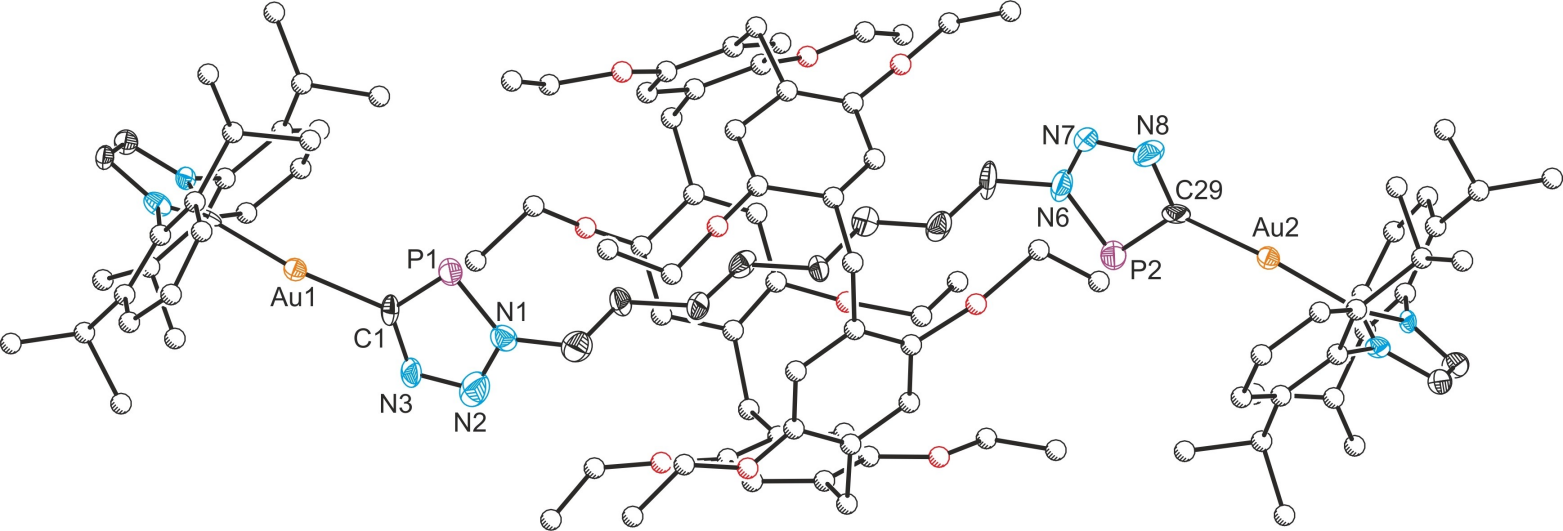
Single crystal XRD structure of **3a**. Anisotropic displacement ellipsoids depicted at 30 % probability. Hydrogen atoms and solvent of crystallization omitted for clarity. Carbon atoms of Dipp substituents and of the **P5A** component pictured as spheres of arbitrary radius.

Comparison of the ^1^H NMR spectra of the [2]rotaxane **3a**, **P5A** and axle **4a** provides indication of the formation of an interlocked molecule (Figure 2). The ^1^H NMR spectrum of rotaxane **3a** (Figure 2B) reveals that three proton resonances (H_a_, H_b_ and H_c_) undergo significant upfield shifts relative to **4a** due to shielding by the **P5A**. These are typical perturbations for [2]rotaxanes based on pillar[5]arene derivatives.^[40]^ By contrast, the proton resonance for the methylene groups immediately adjacent to triazaphosphole moieties (H_d_) is shifted downfield slightly, presumably due to the presence of the large diisopropyl substituents of the stopper preventing **P5A** from moving along the full length of the axle. Also worth noting is that the methylene protons of the ethoxy groups of **P5A** (H_5_) become diasterotopic on formation of the interlocked molecule. This is due to restricted through oxygen‐annulus rotation upon threading of the rotaxane axle which prevents interconversion of the conformers of the planar chiral **P5A** sub‐component.[^37^, ^41^]

**Figure 2:**
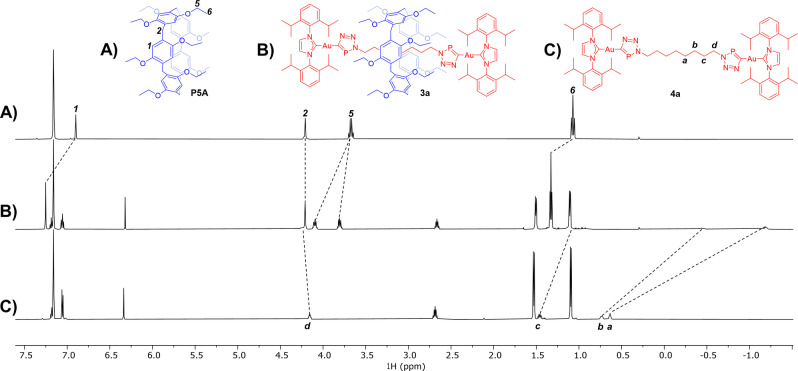
Stacked ^1^H NMR spectra (C_6_D_6_) of: A) macrocycle **P5A**; B) [2]rotaxane **3a**; and C) dumbbell **4a**.

To improve the degree of pseudo‐rotaxane formation and avoid having to use an excess of **P5A**, a new axle was prepared featuring two triazole units. Ogoshi and Li have demonstrated that **P5A** threads triazole‐containing axles with strong binding affinities, due to strong C−H⋅⋅⋅N and C−H⋅⋅⋅O hydrogen bond formation, which can be used to target rotaxanes.[^40^, ^42^, ^43^] Indeed, **P5A** was shown to thread azide **1b** with a greater binding affinity in C_6_D_6_ (K=12500 M^−1^), so an equimolar ratio of the two was used for the synthesis of a rotaxane. Following the same synthetic approach used for the synthesis of **3a**, addition of two equivalents of Au(IDipp)(CP) led to formation of the rotaxane **3b** alongside minor amounts of the non‐interlocked axle **4b** (approx. 10 : 1 ratio of **3b** : **4b**), possessing similar chemical shifts in the ^31^P{^1^H} NMR spectrum at 201.3 and 201.9 ppm, respectively. As with **4a**, **4b** was independently synthesized and characterized by reaction of **1b** with two equivalents of Au(IDipp)(CP) (see Supporting Information for details). The pure rotaxane **3b** could similarly be isolated in good yields (65 %) by chromatographic methods. Single crystal X‐ray diffraction confirmed the formation of a rotaxane (see Supporting Information). The ^1^H NMR spectrum of rotaxane **3b** (Figure 3B) features significantly upfield shifted resonances for the doubly N‐terminated linker axle protons (H_a_ and H_b_), whereas, the proton resonances for the *N*,*C*‐terminated linker (H_e_ and H_f_) are slightly shifted downfield. This indicates that the **P5A** macrocycle predominantly resides on the central doubly N‐terminated linker, shielding the protons contained within the cavity of the macrocycle. Interestingly, the triazole proton (H_c_) resonance undergoes a significant downfield shift. We attribute this to a dominant deshielding effect due to intramolecular hydrogen bonds with the ether groups of the **P5A** macrocyle. Splitting of the **P5A** methylene proton environments (H_5_) upon formation of the [2]rotaxane is also observed, in line with what was recorded for **3a**.

**Figure 3:**
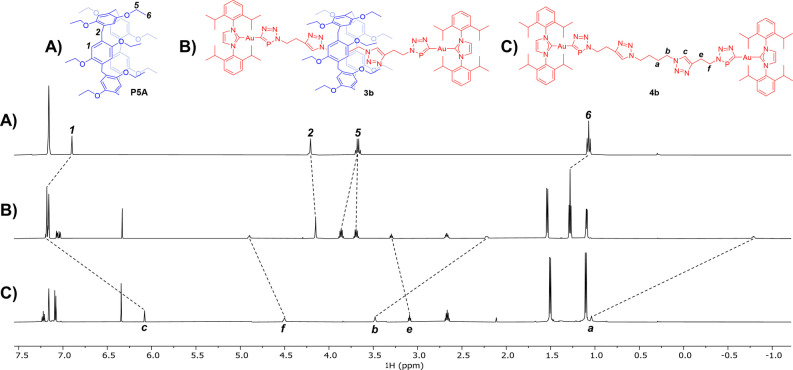
Stacked ^1^H NMR spectra (C_6_D_6_) of: A) macrocycle **P5A**, B) [2]rotaxane **3b**; and C) dumbbell **4b**.

The ^13^C NMR spectra of the two rotaxanes revealed characteristic doublets of the carbon environment in the triazaphosphole moieties with strong ^1^ *J*_C−P_ coupling constants (82.7 Hz and 83.1 Hz for **3a** and **3b**, respectively). The high‐resolution electrospray ionization mass spectra showed peaks corresponding to [M+2H]^2+^ species at *m*/*z*=1173.0573 and 1240.0743 for **3a** and **3b**, respectively.

In conclusion, we have demonstrated the applicability of the cyaphide‐azide click reaction in a new capping approach to synthesize two novel inorganic rotaxanes. The reaction emulates the properties of typical click reactivity in that it is high yielding, highly selective, and is compatible with typical templating techniques for pillar[5]arene based rotaxanes. Importantly, the stabilizing nature of the mechanical bond affords products that can be handled in air and purified by chromatographic methods. Overall, this opens up new avenues to incorporate organometallic stoppers containing low‐valent phosphorus‐moieties into interlocked structures. Further work is focused on expanding the synthetic scope of cyaphide reactivity to allow for strategic design of interlocked structures with desirable properties for catalysis or ion sensing.

## Supporting Information

The authors have cited additional references within the Supporting Information.[^44^, ^45^, ^46^]

## Conflict of interest

The authors declare no conflict of interest.

## Supporting information

As a service to our authors and readers, this journal provides supporting information supplied by the authors. Such materials are peer reviewed and may be re‐organized for online delivery, but are not copy‐edited or typeset. Technical support issues arising from supporting information (other than missing files) should be addressed to the authors.

Supporting Information

Supporting Information

## Data Availability

The data that support the findings of this study are available in the supplementary material of this article.
