# Supplementary material for: An Inorganic Click Reaction for the Synthesis of Interlocked Molecules
Source: Angew Chem Int Ed Engl. 2023 Aug 11;62(38):e202309211. doi: 10.1002/anie.202309211 (PMC10953421; doi:10.1002/anie.202309211)
Supplement: Supplementary file 2 — Supporting Information [file ANIE-62-0-s002.pdf]

Angewandte Chemie
Eine Zeitschrift der Gesellschaft Deutscher Chemiker

Supporting Information

An Inorganic Click Reaction for the Synthesis of Interlocked Molecules

A. Mapp, J. T. Wilmore, P. D. Beer, J. M. Goicoechea**

Contents

1. Experimental section 2

1.1 General experimental methods 2

1.2 Synthesis of reported compounds 3

1.3 NMR Host-Guest binding studies 22

2. Single crystal X-ray diffraction data 23

2.1 X-ray data collection and refinement 23

2.2 X-ray data for **3b** 24

3. References 25

1. Experimental section

1.1 General experimental methods

Synthetic methods. Unless otherwise stated, all reactions and product manipulations were carried out using standard Schlenk-line techniques under an inert atmosphere of argon, or in a dinitrogen filled glovebox (MBraun UNIlab glovebox maintained at < 0.1 ppm H_2O and < 0.1 ppm O_2). Perethylated pillar[5]arene (**P5A**),[38] Mg(^{Dipp}NacNac)(dioxane)(CP)[29] and Au(IDipp)(CP)[29] were synthesized according to the previously reported synthetic procedures. 1,8-diazidooctane (**1a**) and 1,4-diazidobutane were synthesized from the dibromo precursors – 1,8-dibromooctane (Alfa Aesar, 98%) and 1,4-dibromobutane (Alfa Aesar, 98%) – using NaN_3 in DMSO. Toluene (Sigma Aldrich, HPLC grade), hexane (Sigma Aldrich, HPLC grade), and pentane (Sigma Aldrich, HPLC grade) were purified using an MBraun SPS-800 solvent system. $CDCl_3$ (Aldrich, 99.8%) was used as purchased without further purification. C_6D_6 (Aldrich, 99.5%) was degassed dried over CaH_2. All dry solvents were stored under argon in gas-tight ampoules over activated 3 Å molecular sieves.

Characterization techniques. NMR spectra were acquired on a Bruker AVIII 400 MHz NMR spectrometer (^{1}H 400 MHz, ^{31}P 162 MHz), Bruker AVIII 500 MHz NMR spectrometer (^{1}H 500 MHz, ^{13}C 126 MHz, ^{31}P 202 MHz) or a Bruker Avance NEO 600 MHz NMR spectrometer with a broadband helium cryoprobe (^{13}C 151 MHz). ^{1}H and ^{13}C NMR spectra were referenced to the most downfield solvent resonance (^{1}H NMR $CDCl_3$: 7.26 ppm, ^{13}C NMR $CDCl_3$: 77.16; ^{1}H NMR C_6D_6: δ = 7.16 ppm, ^{13}C NMR C_6D_6: δ = 128.06 ppm). ^{31}P NMR spectra were externally referenced to an 85% solution of H_3PO_4 in H_2O. High resolution electrospray ionisation (HR ESI) mass spectrometry were acquired on a Thermo Orbitrap Exactive MS with Waters Acquity Ultraperformance LC system, 90 % MeOH, 0.1 % formic acid buffer, loop injection/direct infusion.

1.2 Synthesis of reported compounds

1.2.1 Synthesis of $1b^{Br}$

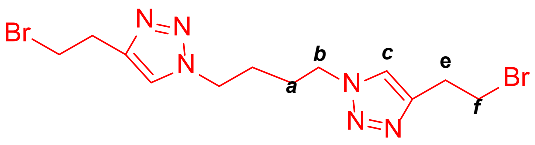

TBTA (56.0 mg, 0.11 mmol) was added to a solution of 1,4-diazidobutane (400 mg, 2.85 mmol, 1.0 equiv.) and 4-bromobutyne (797 mg, 6.00 mmol, 2.1 equiv.) in chloroform (10 mL) and stirred for 1 h at room temperature. $[Cu(CH_3CN)_4]PF_6$ (40.0 mg, 0.105 mmol) was added and the reaction mixture was stirred for 24 h at room temperature. The reaction mixture was poured onto hexane and the precipitate was collect. This was used without further purification. Yield (720 mg, 1.77 mmol, 62%).

^{1}H NMR (600 MHz, $CDCl_3$): δ (ppm) 7.49 (s, br, 2H; H_c), 4.38 (s, br, 4H; H_b), 3.66 (s, br, 4H; H_f), 3.30 (s, br, 4H; H_e), 1.94 (s, br, 4H; H_a).

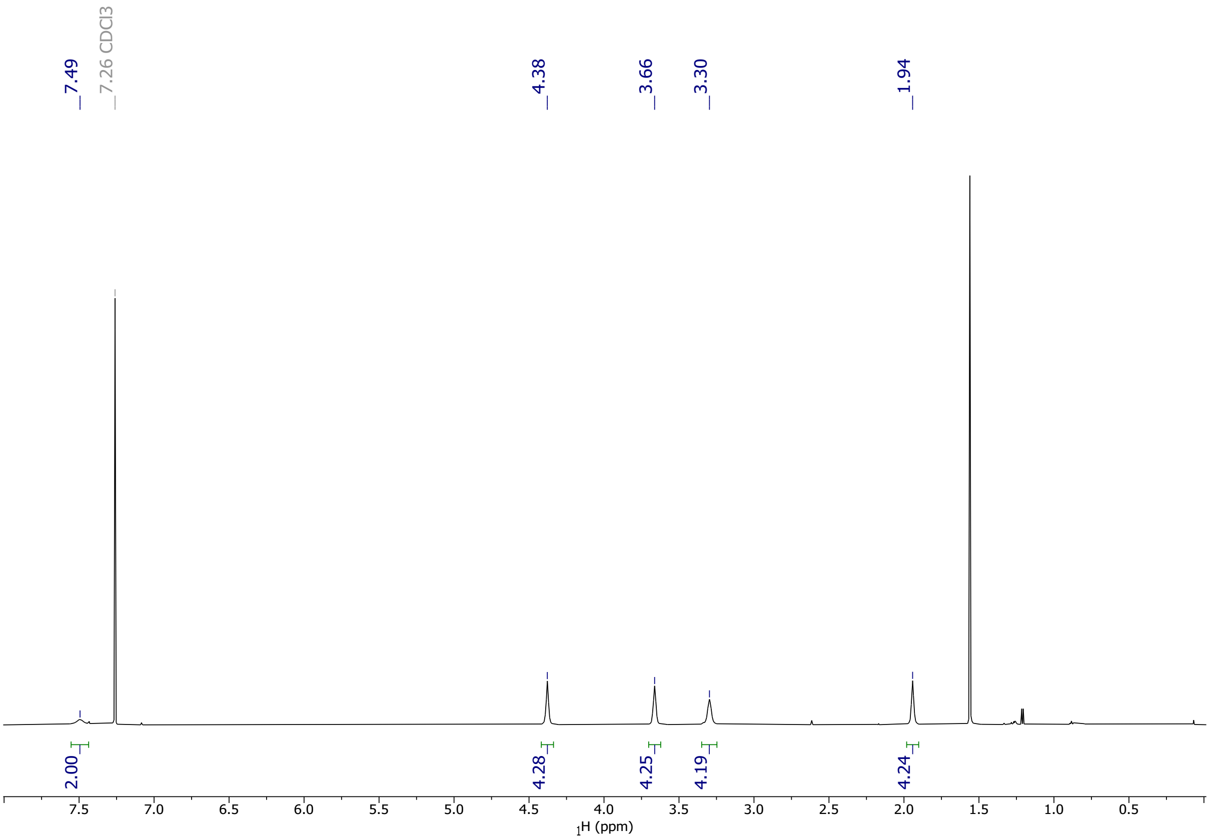

Figure S1. ^{1}H NMR (600 MHz) spectrum of **$1b^{Br}$** in $CDCl_3$.

1.2.2 Synthesis of 1b

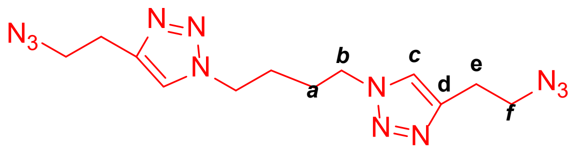

NaN_3 (458 mg, 7.05 mmol, 4.0 equiv.) and trace NaI was added to a solution of **1b^{Br}** (715 mg, 1.76 mmol, 1.0 equiv.) in DMSO (20 mL) and stirred overnight at room temperature. The solution was diluted with H_2O and the product was extracted with DCM (3 × 60 mL). The organic extracts were combined and washed with brine (3 × 60 mL), then dried over Na_2SO_4. The solvent was removed *in vacuo* and the crude product was purified on a silica gel column using MeOH/DCM (1:9 v/v) to afford product **1b** as a white powder. Yield: 324 mg, 0.981 mmol, 56%.

^{1}H NMR (600 MHz, $CDCl_3$): δ (ppm) 7.37 (s, 2H; H_c), 4.36 (m, 4H; H_b), 3.62 (t, $^3J_{H\text{–}H}$ = 6.7 Hz, 4H; H_f), 3.00 (t, $^3J_{H\text{–}H}$ = 6.7 Hz, 4H; H_e), 1.93 (m, 4H; H_a).

^{13}C NMR (151 MHz, $CDCl_3$): δ (ppm) 144.64 (C_d), 121.89 (C_c), 50.75 (C_f), 49.41 (C_b), 27.26 (C_a), 25.92 (C_e).

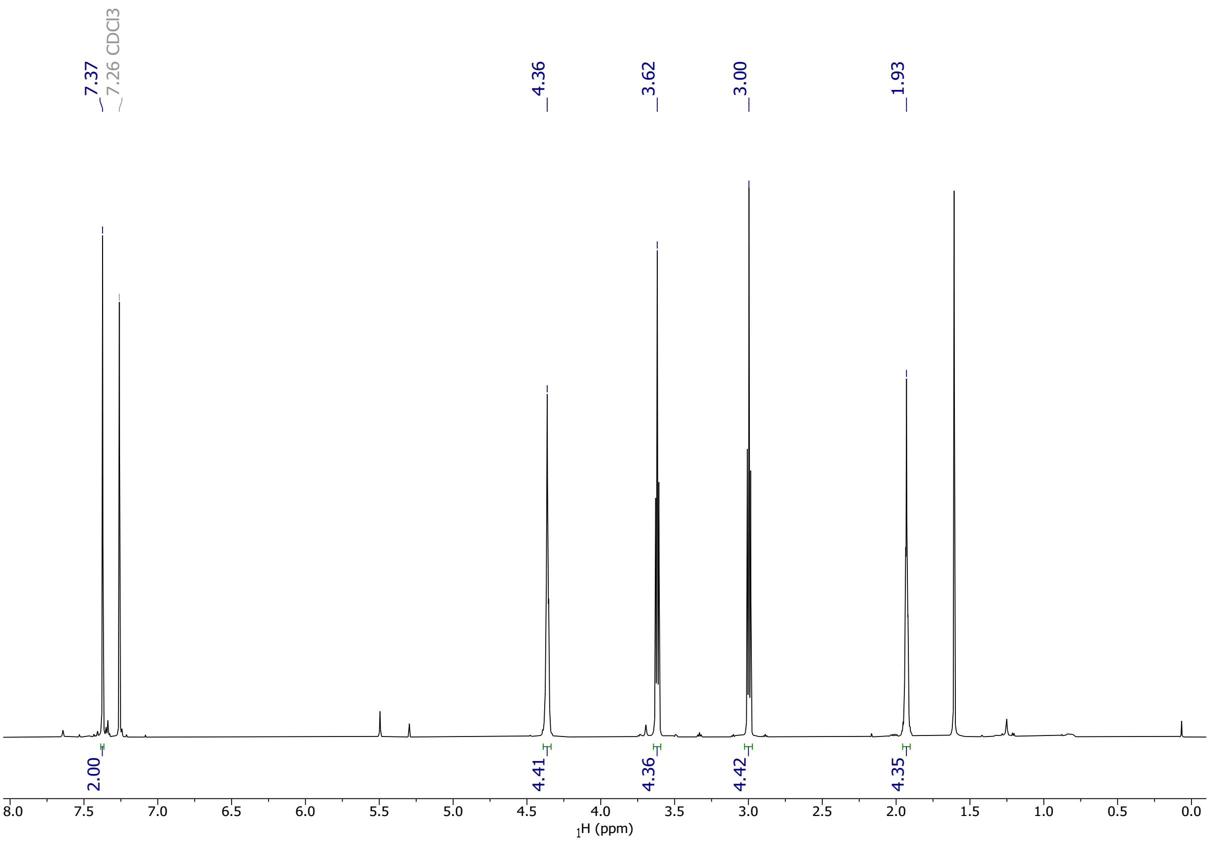

Figure S2. ^{1}H NMR (600 MHz) spectrum of **1b** in $CDCl_3$.

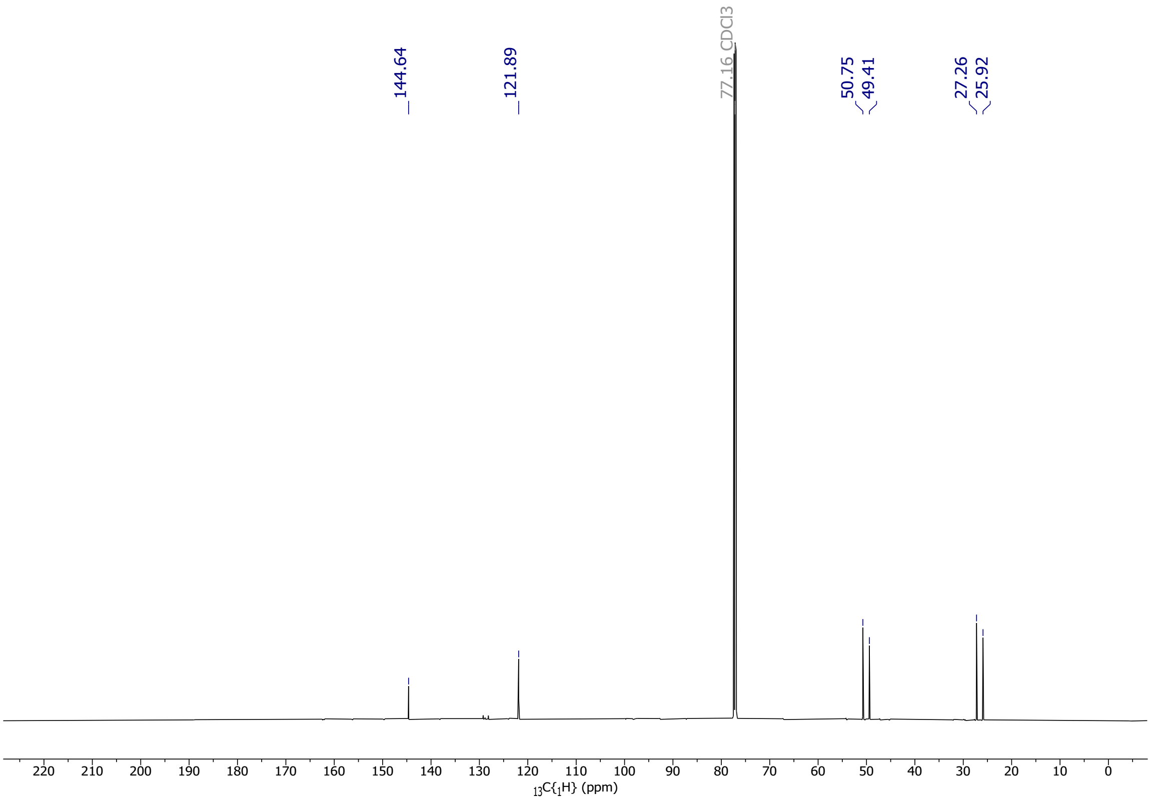

Figure S3. $^{13}C\{^{1}H\}$ NMR (151 MHz) spectrum of **1b** in $CDCl_3$.

1.2.3 Synthesis of 3a

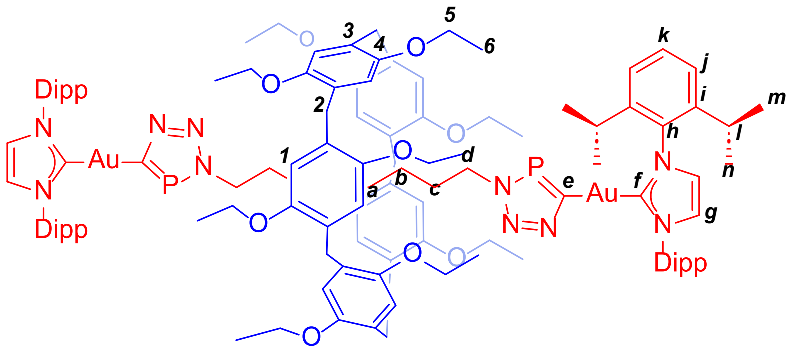

P5A (103 mg, 0.115 mmol, 5.0 equiv.) was dissolved in a solution of **1a** (4.5 mg, 22.9 μmol, 1.0 equiv.) in benzene (2 mL). Au(IDipp)(CP) (30.3 mg, 48.2 μmol, 2.10 equiv.) was added to the solution and the reaction mixture was stirred overnight at room temperature. The precipitate was isolated by filtration and dried *in vacuo*. The crude product was purified on a silica gel column using EtOAc/hexane (3:7 v/v) to afford product **3a** as a white residue. Yield: 20.5 mg, 8.71 μmol, 38%.

^{1}H NMR (600 MHz, C_6D_6): δ (ppm) 7.26 (s, 10H; H_1), 7.19 (m, 4H; H_k), 7.06 (*pseudo*-triplet, $^3J_{H–H}$ = 7.8 Hz, 8H; H_j), 6.31 (s, 4H; H_g), 4.26 (br, 4H; H_d), 4.22 (s, 10H; H_2), 4.10 (m, 10H; H_5), 3.81 (m, 10H; H_5), 2.67 (sept, $^3J_{H–H}$ = 6.9 Hz, 8H; H_l), 1.51 (dd, $^3J_{H–H}$ = 6.9 Hz, 24H; $H_{n/m}$), 1.33 (t, $^3J_{H–H}$ = 6.9 Hz, 30H; H_6) 1.10 (dd, $^3J_{H–H}$ = 6.9 Hz, 24H; $H_{n/m}$), 1.08 (q, $^3J_{H–H}$ = 7.7 Hz, 4H; H_c),* –0.44 (br, 4H; H_b), –1.19 (br, 4H; H_a).

^{13}C NMR (151 MHz, C_6D_6): δ (ppm) 206.74 (d, $^1J_{C–P}$ = 82.7 Hz; C_e), 196.17 (d, $^3J_{C–P}$ = 10.4 Hz; C_f), 150.33 (C_4), 145.85 (C_i), 134.75 (C_h), 130.73 (C_k), 128.76 (C_3), 124.28 (d, J = 3.7 Hz; C_j)*, 122.75 (C_g), 114.48 (C_1), 63.74 (C_5), 52.43 (d, $^2J_{C–P}$ = 9.6 Hz; C_d), 34.61 (C_c), 29.92 (C_2), 29.11 (C_l), 28.51 (C_a), 26.58 (C_b), 24.88 ($C_{n/m}$), 23.97 ($C_{n/m}$), 15.81 (C_6).

^{31}P NMR (243 MHz, C_6D_6): δ (ppm) 198.6.

HRMS (ESI, +ve ion mode): *m*/*z* calcd. for $C_{119}H_{158}Au_2N_{10}O_{10}P_2$+2H$^+$: 1173.0573 [*M*+2H]$^{2+}$; found 1173.0557.

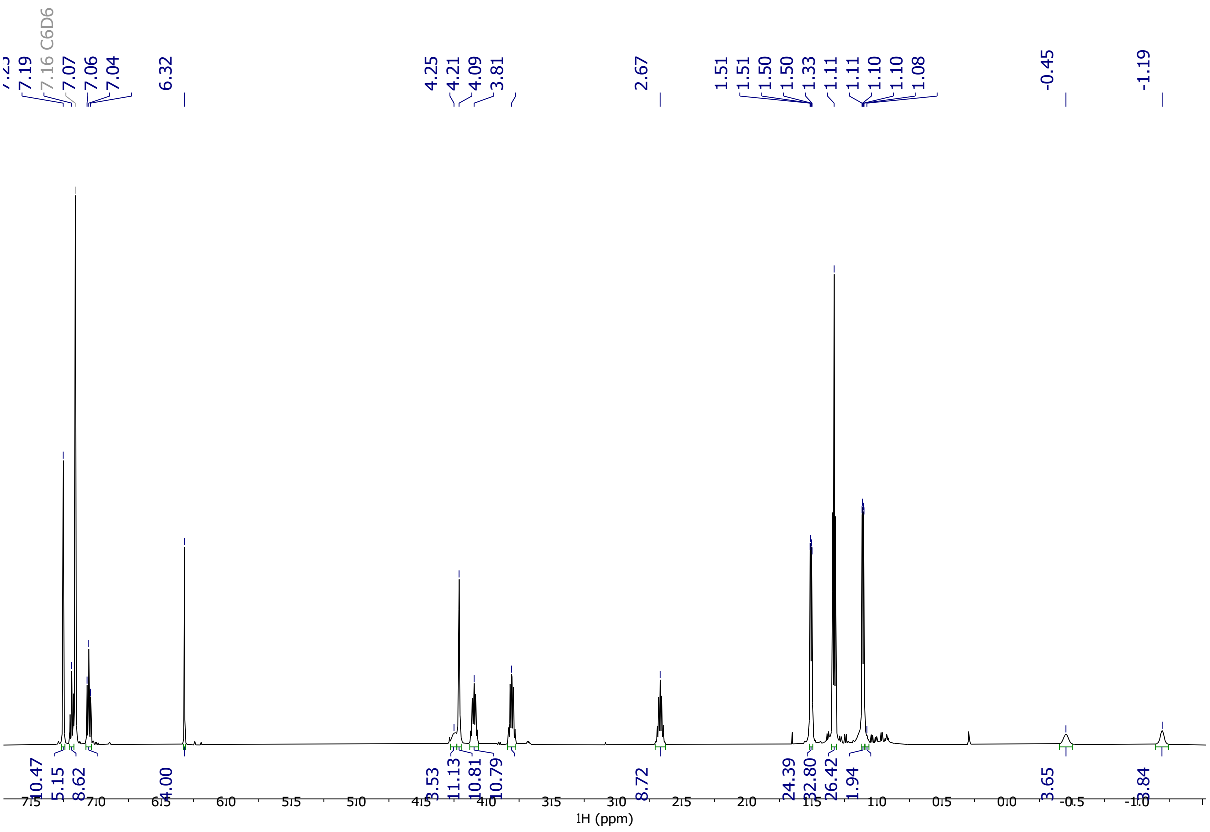

Figure S4. ^{1}H NMR (600 MHz) spectrum of **3a** in C_6D_6.

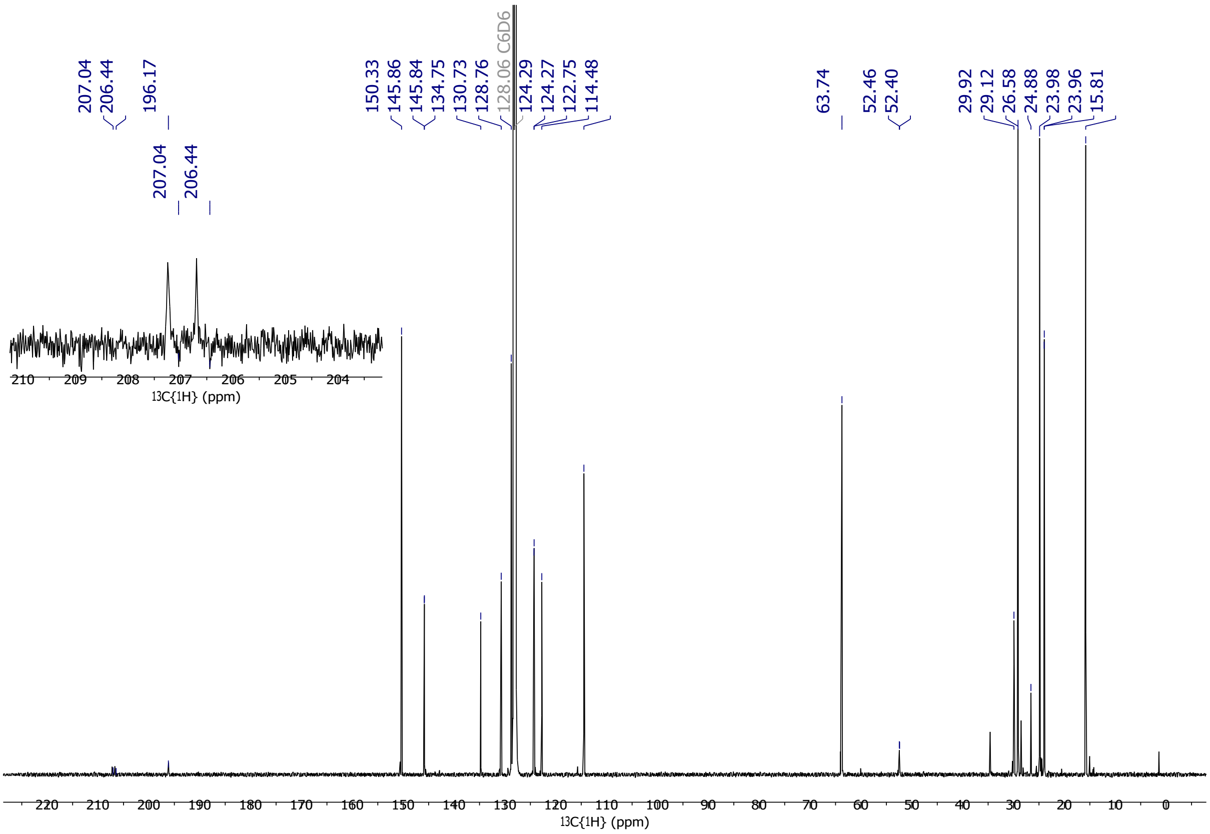

Figure S5. $^{13}C\{^{1}H\}$ NMR (151 MHz) spectrum of **3a** in C_6D_6.

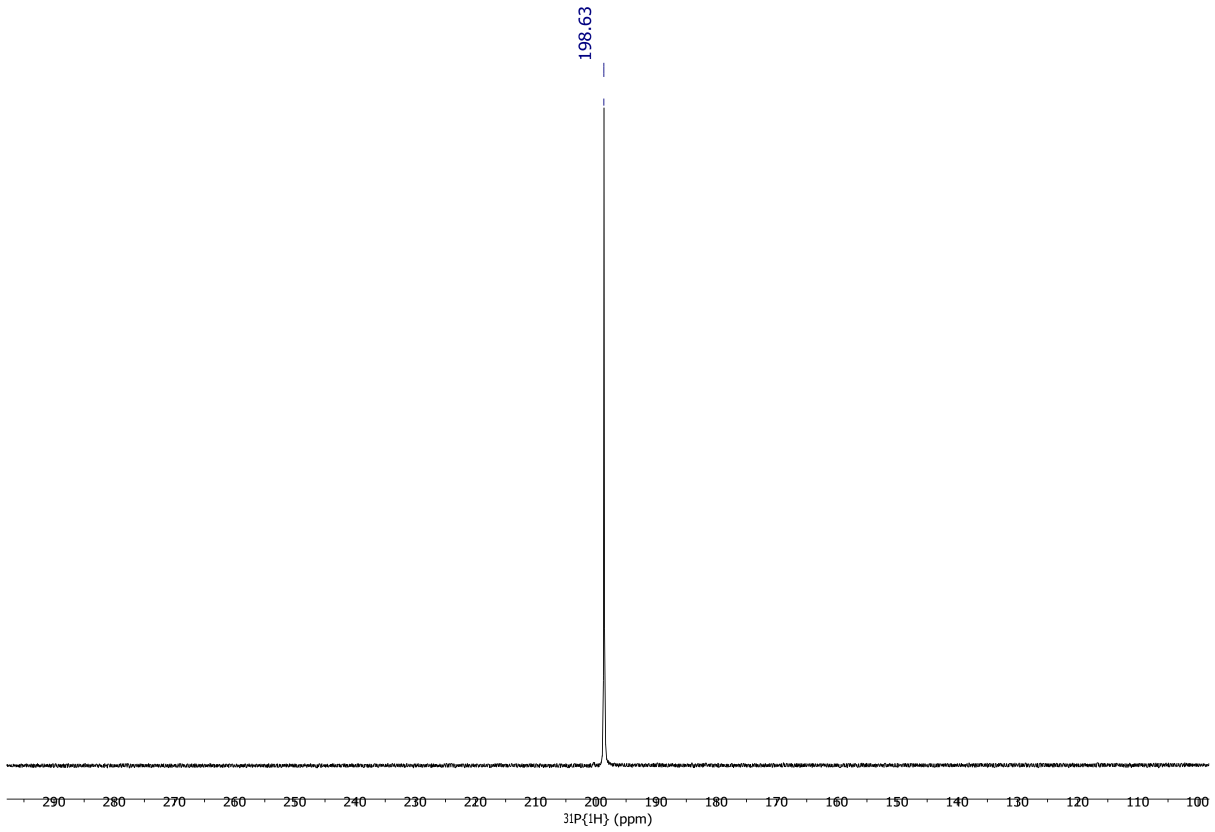

Figure S6. $^{31}P\{^{1}H\}$ NMR (243 MHz) spectrum of **3a** in C_6D_6.

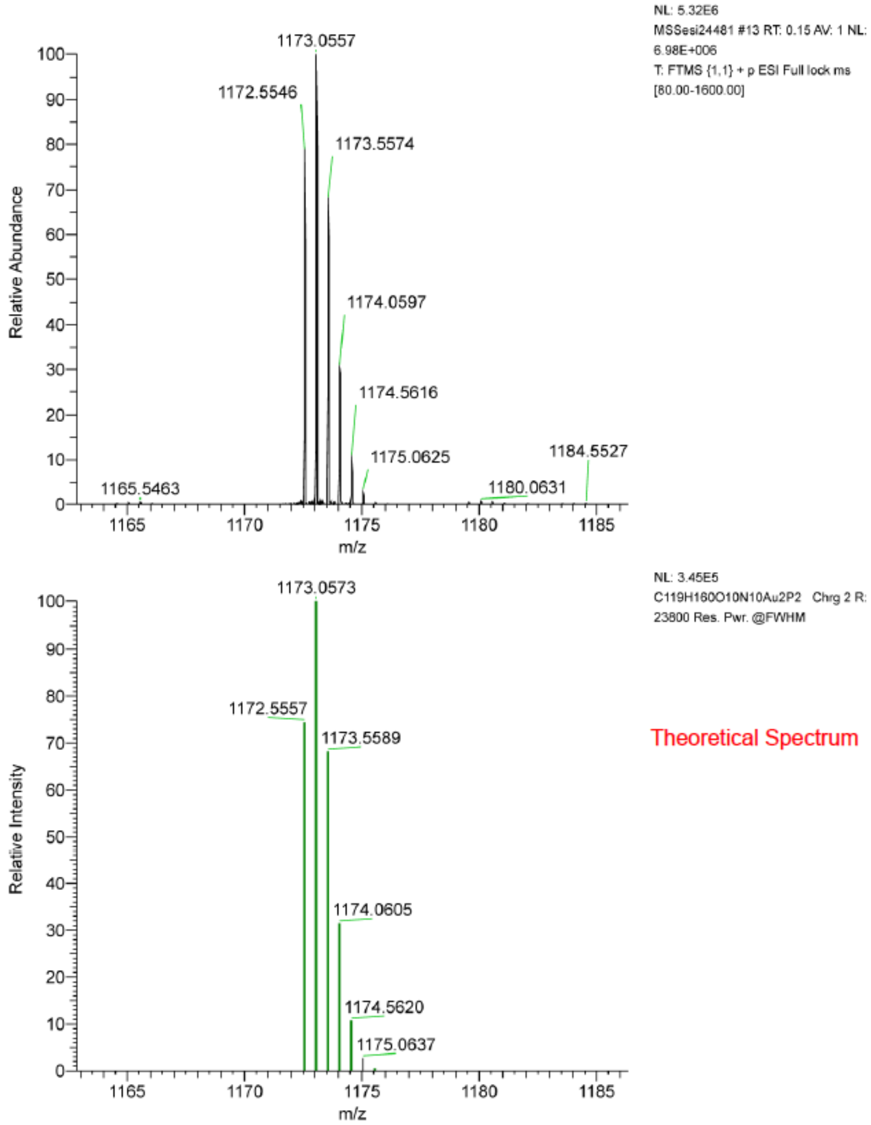

Figure S7. Top: high-resolution mass spectrum of **3a** showing the $[M+2H]^{2+}$ ion at *m*/*z* 1173.0557. Bottom: theoretical spectrum.

1.2.4 Synthesis of 3b

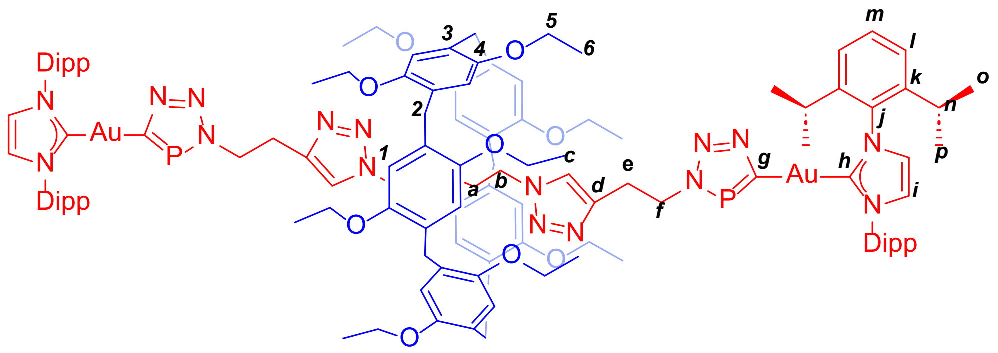

P5A (40.6 mg, 45.1 μmol, 1.0 equiv.) was dissolved in a solution of **1b** (14.9 mg, 45.1 μmol, 1.0 equiv.) in benzene (2 mL). Au(IDipp)(CP) (59.5 mg, 94.7 μmol, 2.10 equiv.) was added to the solution and the reaction mixture was stirred overnight at room temperature. The solvent was removed *in vacuo* and the crude product was purified on a silica gel column using EtOAc/hexane (1:1 v/v) to afford product **3b** as a white powder. Yield: 72.9 mg, 29.3 μmol, 65%. Crystals were grown by slow diffusion of hexane into a saturated toluene solution of **3b**.

^{1}H NMR (600 MHz, C_6D_6): δ (ppm) 7.18 (m; H_1, H_c, H_m),* 7.05 (ddd, 8H; H_j), 6.33 (s, 4H; H_i), 4.90 (dt, $^{3}J_{H–H}$ = 7.3 Hz, $^{3}J_{H–P}$ = 3.6 Hz, 4H; H_f), 4.15 (s, 10H; H_2), 3.86 (m, 10H; H_5), 3.69 (m, 10H; H_5), 3.30 (t, $^{3}J_{H–H}$ = 7.3 Hz 4H; H_e), 2.67 (sept, $^{3}J_{H–H}$ = 6.9 Hz, 8H; H_n), 2.22 (m, 4H; H_b), 1.54 (d, $^{3}J_{H–H}$ = 6.9 Hz, 24H; $H_{o/p}$), 1.28 (t, $^{3}J_{H–H}$ = 6.9 Hz, 30H; H_6) 1.10 (dd, $^{3}J_{H–H}$ = 6.9 Hz, 24H; $H_{o/p}$), –0.79 (m, 4H; H_a). *Overlapping peaks prevented integration and multiplet analysis.

^{13}C NMR (151 MHz, C_6D_6): δ (ppm) 207.36 (d, $^{1}J_{C–P}$ = 83.1 Hz; C_g), 195.95 (d, $^{3}J_{C–P}$ = 11.0 Hz; C_h), 150.31 (C_4), 145.84 (C_k), 143.15 (C_d), 134.72 (C_j), 130.79 (C_m), 129.19 (C_3), 124.29 (C_l),* 122.77 (C_i), 115.06 (C_1), 64.10 (C_5), 51.00 (d, $^{2}J_{C–P}$ = 10.1 Hz; C_f), 48.62 (C_b), 29.86 (C_2), 29.81 (C_e), 29.14 (C_n) 25.11 (C_a), 24.92 ($C_{o/p}$), 23.97 ($C_{o/p}$), 15.63 (C_6).

^{31}P NMR (243 MHz, C_6D_6): δ (ppm) 201.3.

HRMS (ESI +ve ion mode): *m*/*z* calcd. for $C_{123}H_{160}Au_2N_{16}O_{10}P_2$+2H$^+$: 1240.0733 [$M$+2H]$^{2+}$; found 1240.0743.

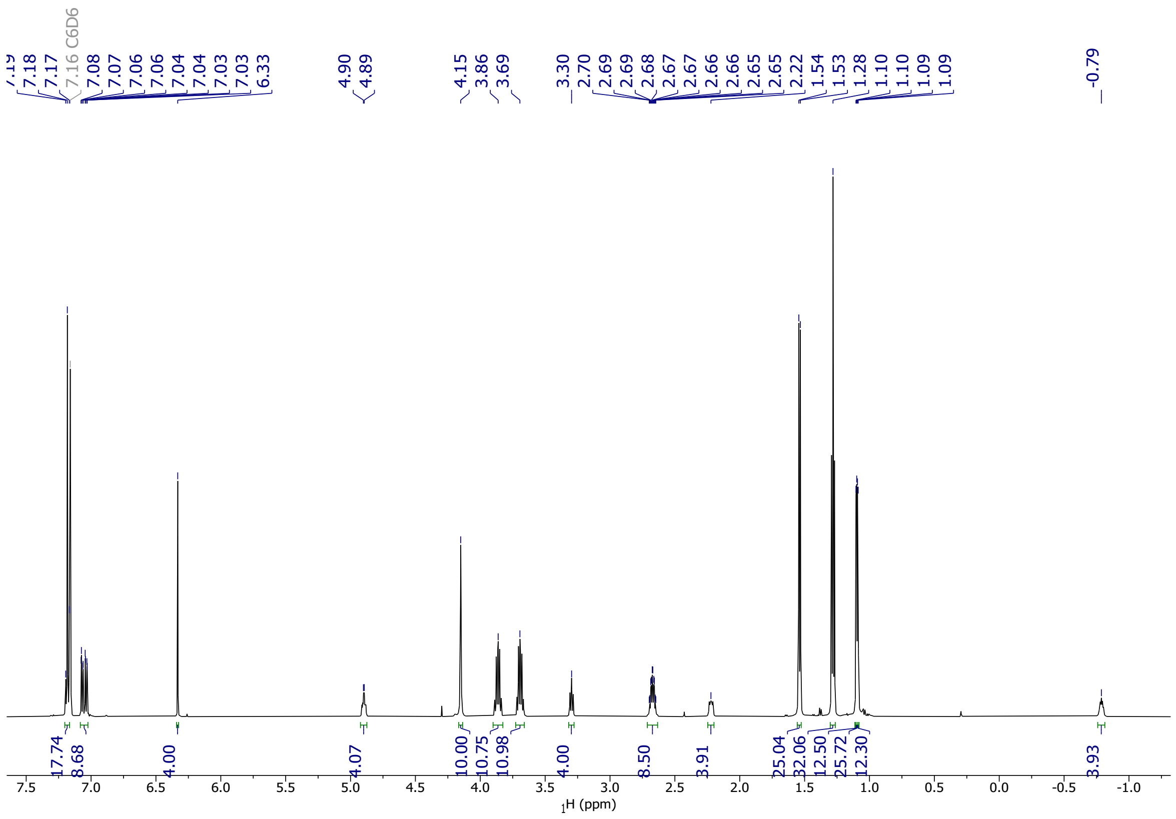

Figure S8. ^{1}H NMR (600 MHz) spectrum of **3b** in C_6D_6.

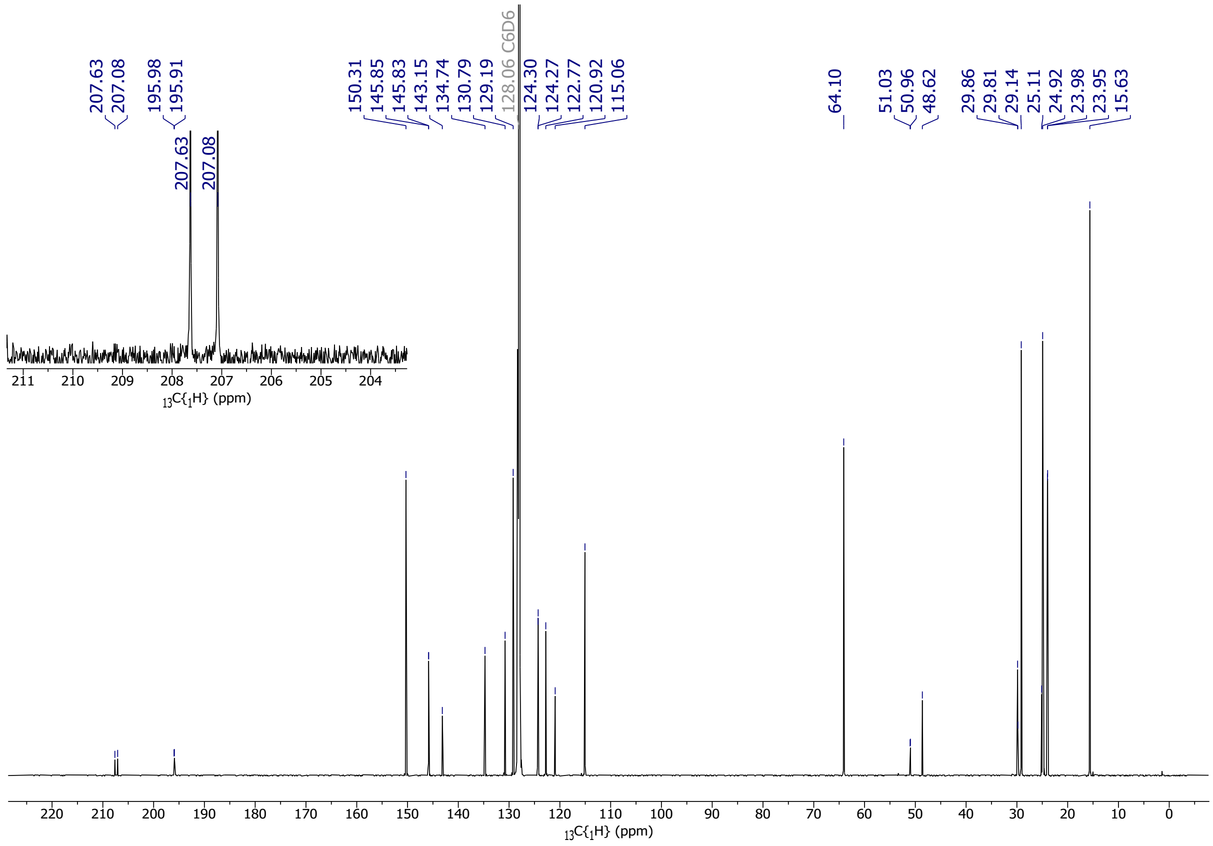

Figure S9. $^{13}C\{^{1}H\}$ NMR (151 MHz) spectrum of **3b** in C_6D_6.

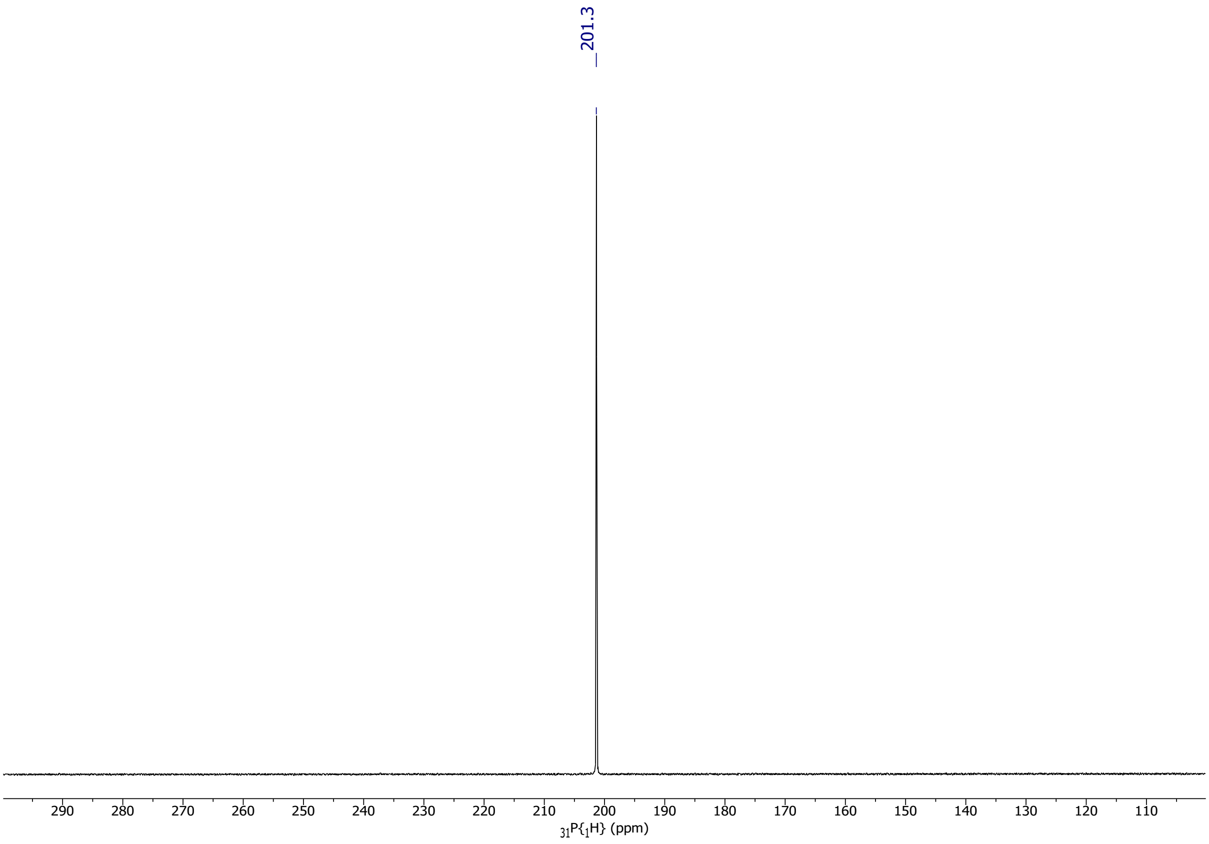

Figure S10. $^{31}P\{^{1}H\}$ NMR (243 MHz) spectrum of **3b** in C_6D_6.

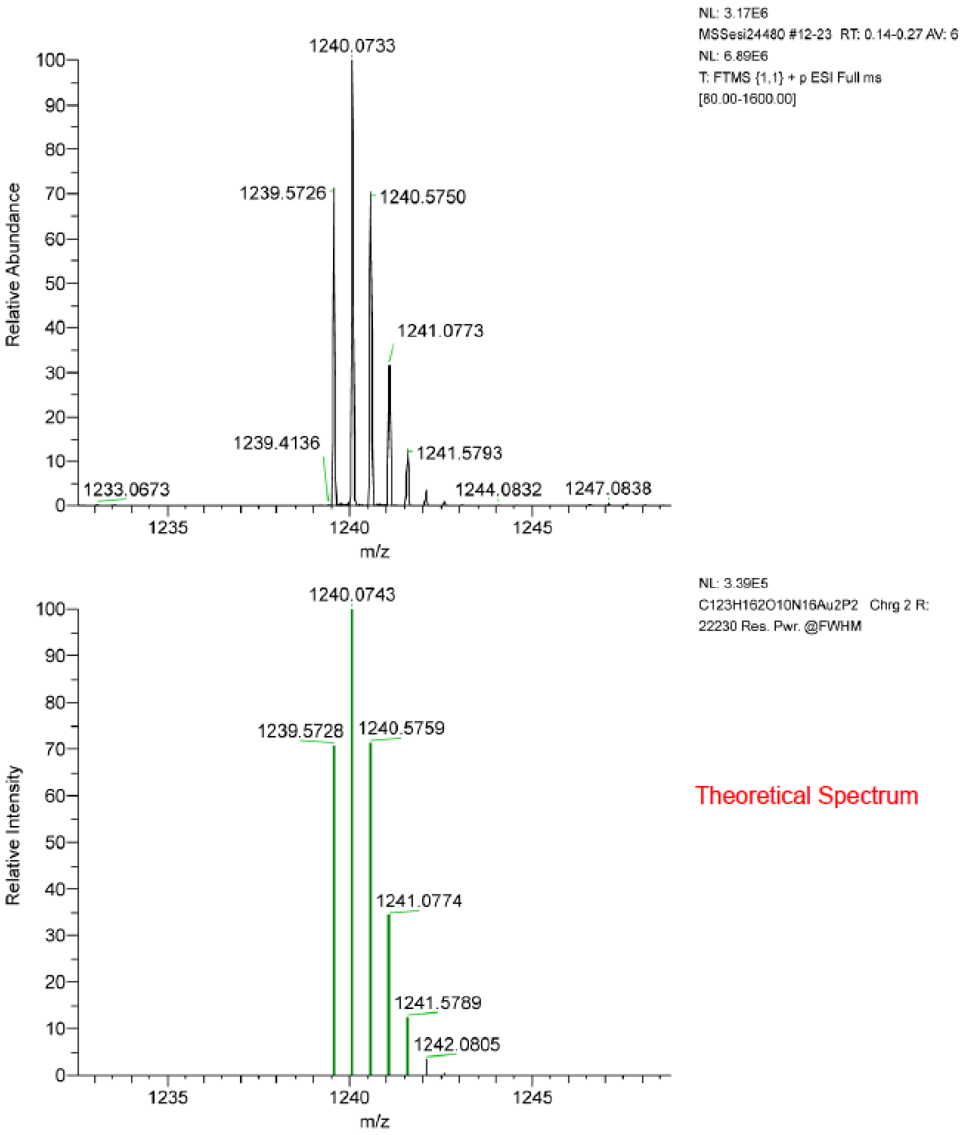

Figure S11. Top: high-resolution mass spectrum of **3b** showing the $[M+2H]^{2+}$ ion at *m*/*z* 1240.0733. Bottom: theoretical spectrum.

1.2.5 Synthesis of 4a

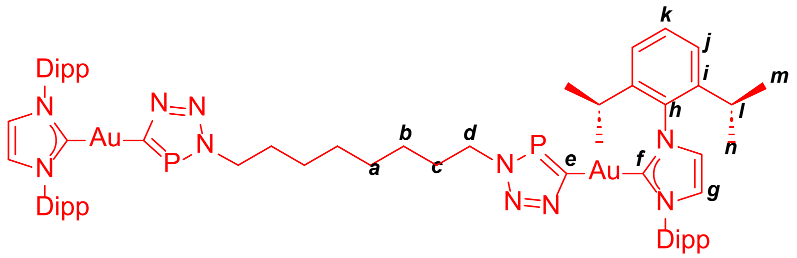

Au(IDipp)(CP) (29.6 mg, 47.1 µmol, 2.1 equiv.) was added to a solution of **1a** (4.40 mg, 22.4 µmol, 1.0 equiv.) in benzene (0.5 mL) and stirred overnight at room temperature. The solution was concentrated and layered with hexane (3 mL). The resulting crystals were isolated by filtration, washed with hexane (3 × 1 mL) and pentane (3 × 1 mL), then dried *in vacuo*. Yield: 23.1 mg, 15.9 µmol, 71%.

^{1}H NMR (400 MHz, C_6D_6): δ (ppm) 7.19 (m, 4H; H_k), 7.06 (d, $^3J_{H-H}$ = 7.8 Hz, 8H; H_j), 6.34 (s, 4H; H_g), 4.16 (m, 4H; H_d), 2.69 (sept, $^3J_{H-H}$ = 6.9 Hz, 8H; H_l), 1.53 (d, $^3J_{H-H}$ = 6.9 Hz, $H_{n/m}$), 1.46 (q, $^3J_{H-H}$ = 7.7 Hz, 4H; H_c), 1.10 (d, $^3J_{H-H}$ = 6.9 Hz, 24H; $H_{n/m}$), 0.74 (m, 4H; H_b), 0.63 (m, 4H; H_a).

^{13}C NMR (151 MHz, C_6D_6): δ (ppm) 207.18 (d, $^1J_{C-P}$ = 82.8 Hz; C_e), 196.39 (d, $^3J_{C-P}$ = 10.9 Hz; C_f), 145.85 (C_i), 134.76 (C_h), 130.73 (C_k), 124.28 (C_j), 122.71 (C_g), 51.43 (d, $^2J_{C-P}$ = 9.6 Hz; C_d), 32.80 (d, $^3J_{C-P}$ = 2.7 Hz; C_c), 29.15 (C_l),* 28.98 (C_a),* 26.66 (C_b), 24.92 ($C_{n/m}$), 23.99 (Dipp $C_{n/m}$). *Assigned based on relative intensities.

^{31}P{^{1}H} NMR (162 MHz, C_6D_6): δ (ppm) 199.9 ppm.

HRMS (ESI, +ve ion mode): *m*/*z* calcd. for $C_{64}H_{88}Au_2N_{10}P_2+H^+$: 1453.6072 [*M*+H]$^+$; found 1453.6032.

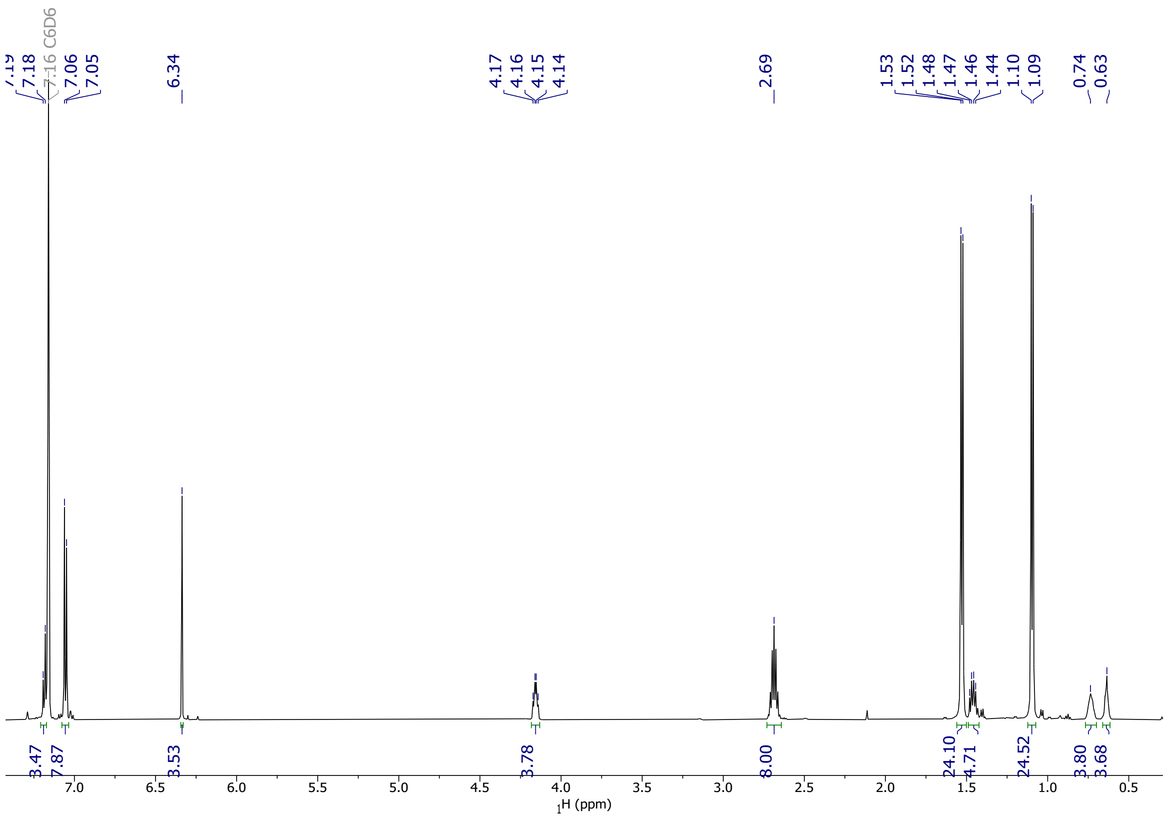

Figure S12. ^{1}H NMR (600 MHz) spectrum of **4a** in C_6D_6.

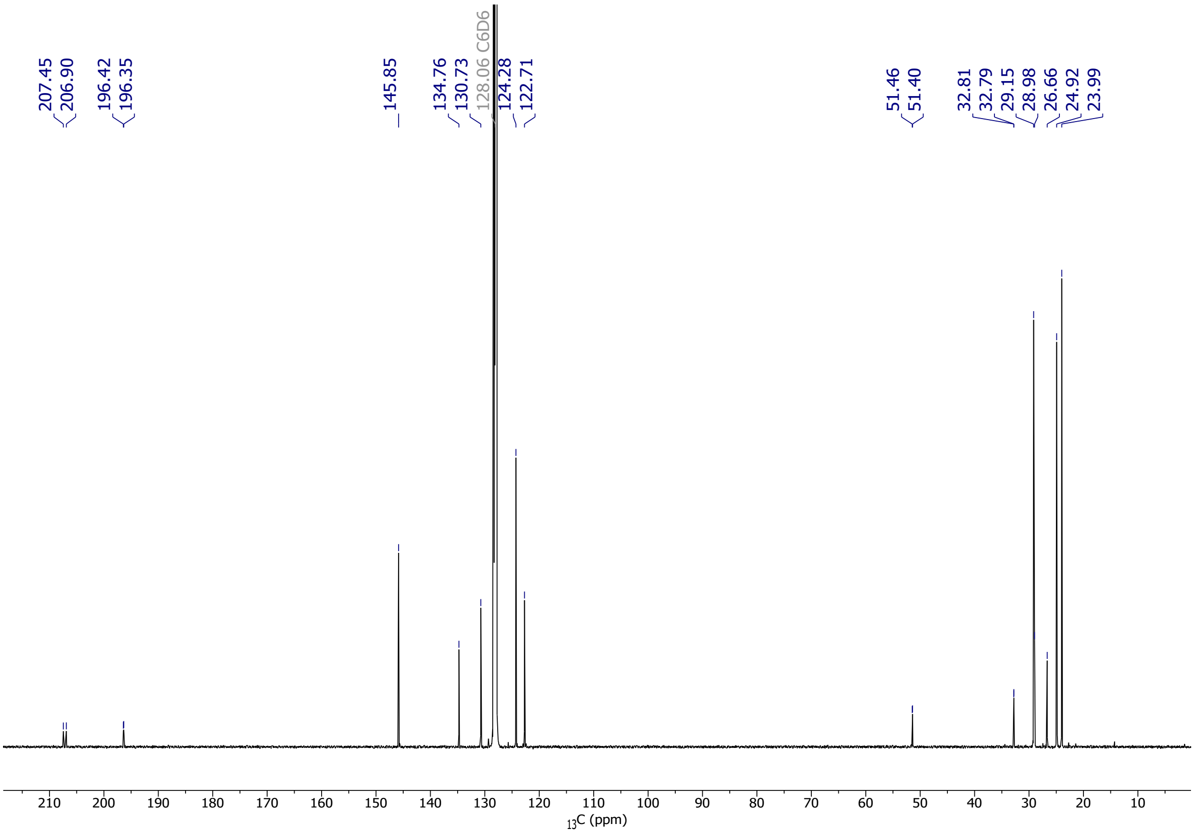

Figure S13. ^{13}C{^{1}H} NMR (151 MHz) spectrum of **4a** in C_6D_6.

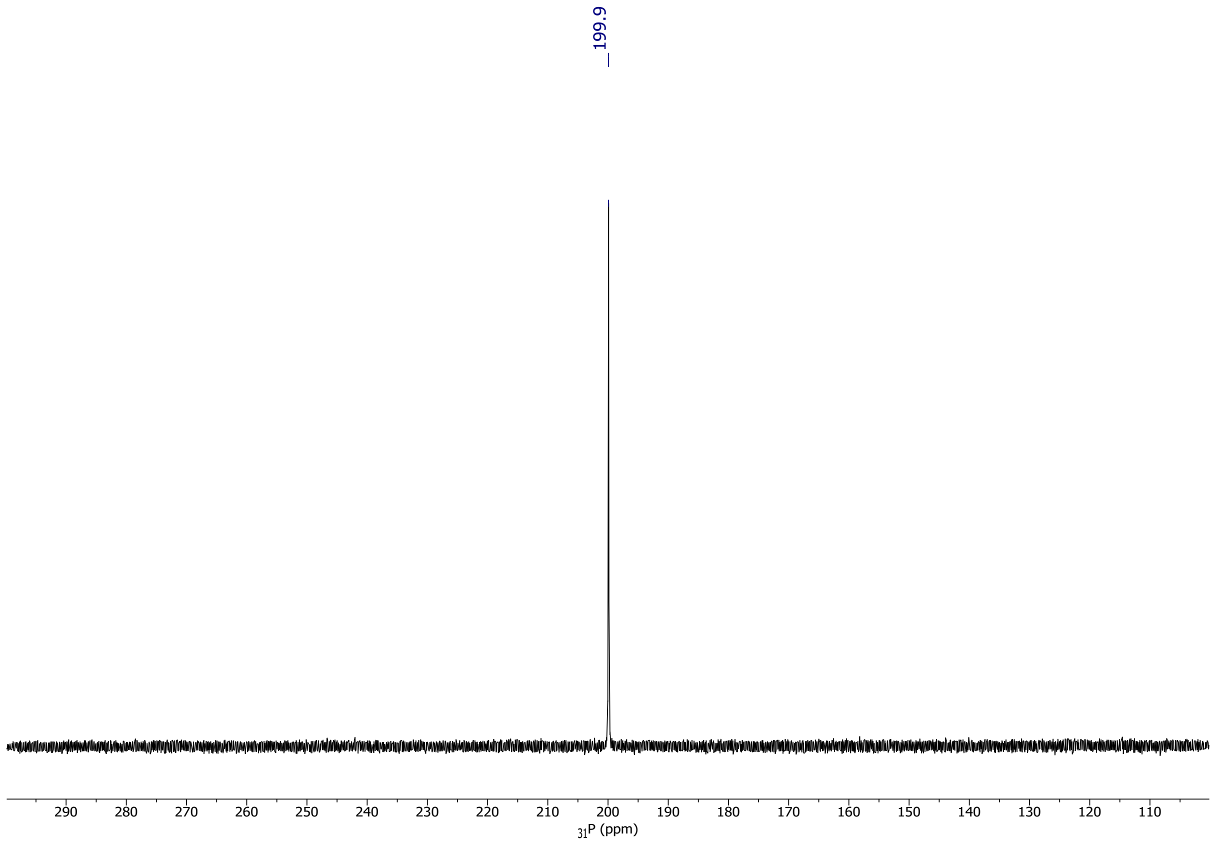

Figure S14. $^{31}P\{^{1}H\}$ NMR (243 MHz) spectrum of **4a** in C_6D_6.

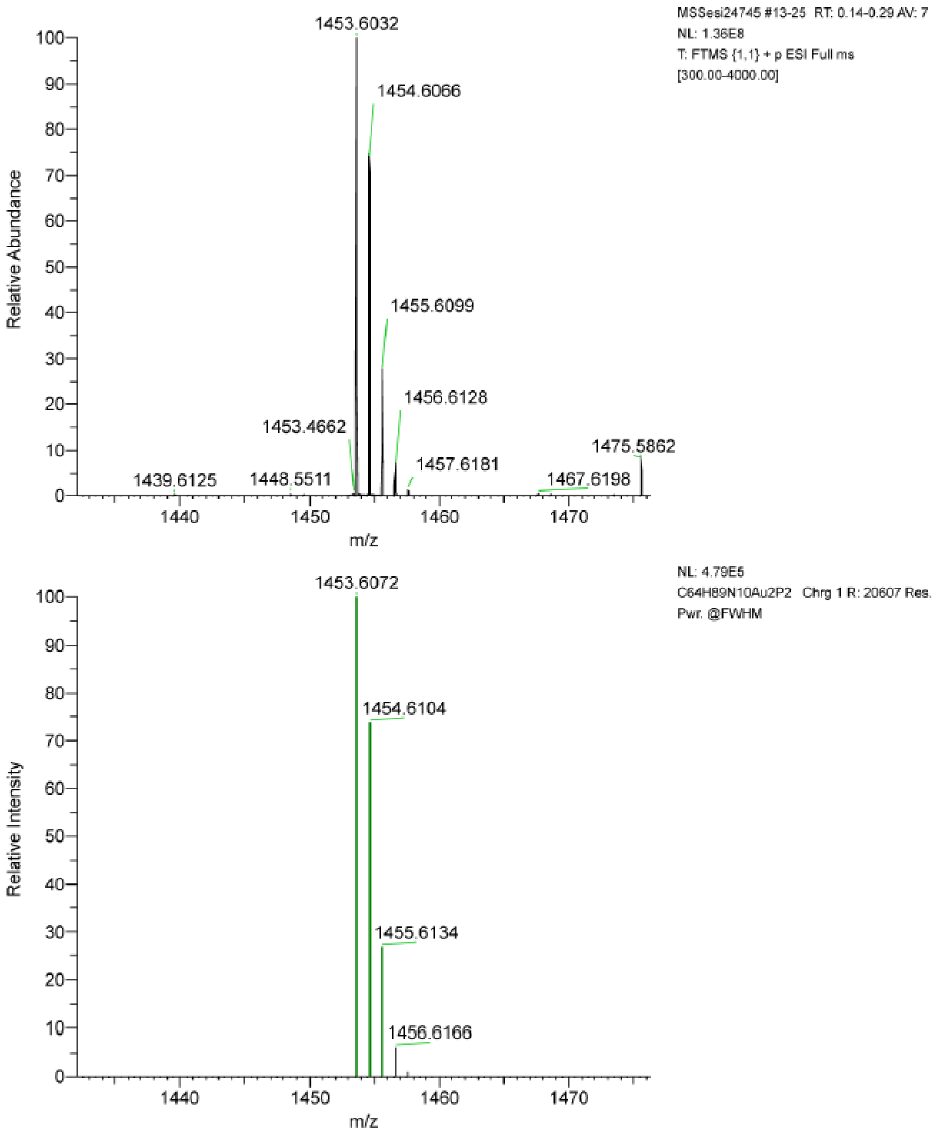

Figure S15. Top: high-resolution mass spectrum of **4a** showing the $[M+H]^+$ ion at *m*/*z* 1453.6032. Bottom: theoretical spectrum.

1.2.6 Synthesis of 4b

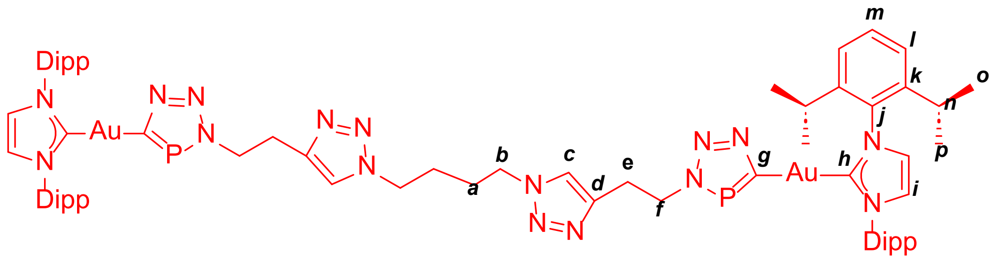

Au(IDipp)(CP) (19.2 mg, 30.5 µmol, 2.1 equiv.) was added to a solution of **1b** (4.8 mg, 14.5 µmol, 1.0 equiv.) in benzene (0.5 mL) and stirred overnight at room temperature. The solution was concentrated and stored at –35 ºC for 7 days to yield yellow crystals. The supernatant was removed, and the crystals were washed with pentane (3 × 1 mL) then dried *in vacuo*. Yield: 17.9 mg, 11.3 µmol, 78%.

^{1}H NMR (600 MHz, C_6D_6): δ (ppm) 7.22 (t, $^3J_{H–H}$ = 7.8 Hz, 4H; H_m), 7.09 (d, $^3J_{H–H}$ = 7.8 Hz, 8H; H_l), 6.34 (s, 4H; H_i), 6.08 (s, 2H; H_c), 4.51 (dt, $^3J_{H–H}$ = 6.4 Hz, $^3J_{H–P}$ = 3.8 Hz, 4H; H_f), 3.48 (m, 4H; H_b), 3.09 (t, $^3J_{H–H}$ = 6.4 Hz, 4H; H_e), 2.67 (sept, $^3J_{H–H}$ = 6.9 Hz, 8H; H_n), 1.51 (d, $^3J_{H–H}$ = 6.9 Hz, 24H; $H_{o/p}$), 1.10 (d, $^3J_{H–H}$ = 6.9 Hz, 24H; $H_{o/p}$), 1.04 (m, 4H; H_a).

^{13}C NMR (151 MHz, C_6D_6): δ (ppm) 207.49 (d, $^1J_{C–P}$ = 83.3 Hz; C_g), 195.80 (d, $^3J_{C–P}$ = 10.9 Hz; C_h), 145.81 (C_k), 144.05 (C_d), 134.68 (C_j), 130.87 (C_m), 124.36 (C_l), 122.83 (C_i), 121.96 (C_c) 50.95 (d, $^3J_{C–P}$ = 9.4 Hz; C_f), 48.34 (C_b), 29.41 (C_e), 29.15 (C_n), 26.49 (C_a), 24.90 ($C_{o/p}$), 23.96 ($C_{o/p}$).

^{31}P NMR (243 MHz, C_6D_6): δ (ppm) 201.9.

HRMS (ESI, +ve ion mode): *m*/*z* calcd. for $C_{68}H_{90}Au_2N_{16}P_2+H^+$: 1587.6413 [*M*+H]$^+$; found 1587.6388.

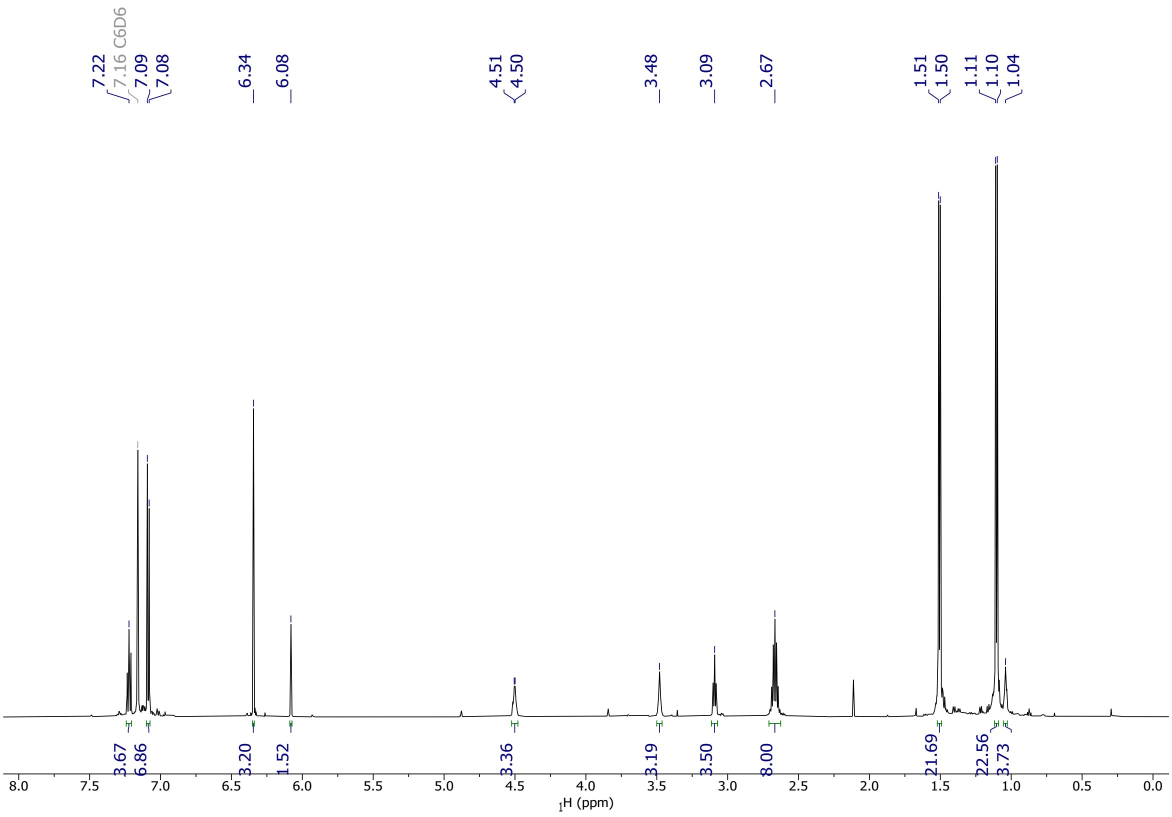

Figure S16. ^{1}H NMR (600 MHz) spectrum of **4b** in $CDCl_3$.

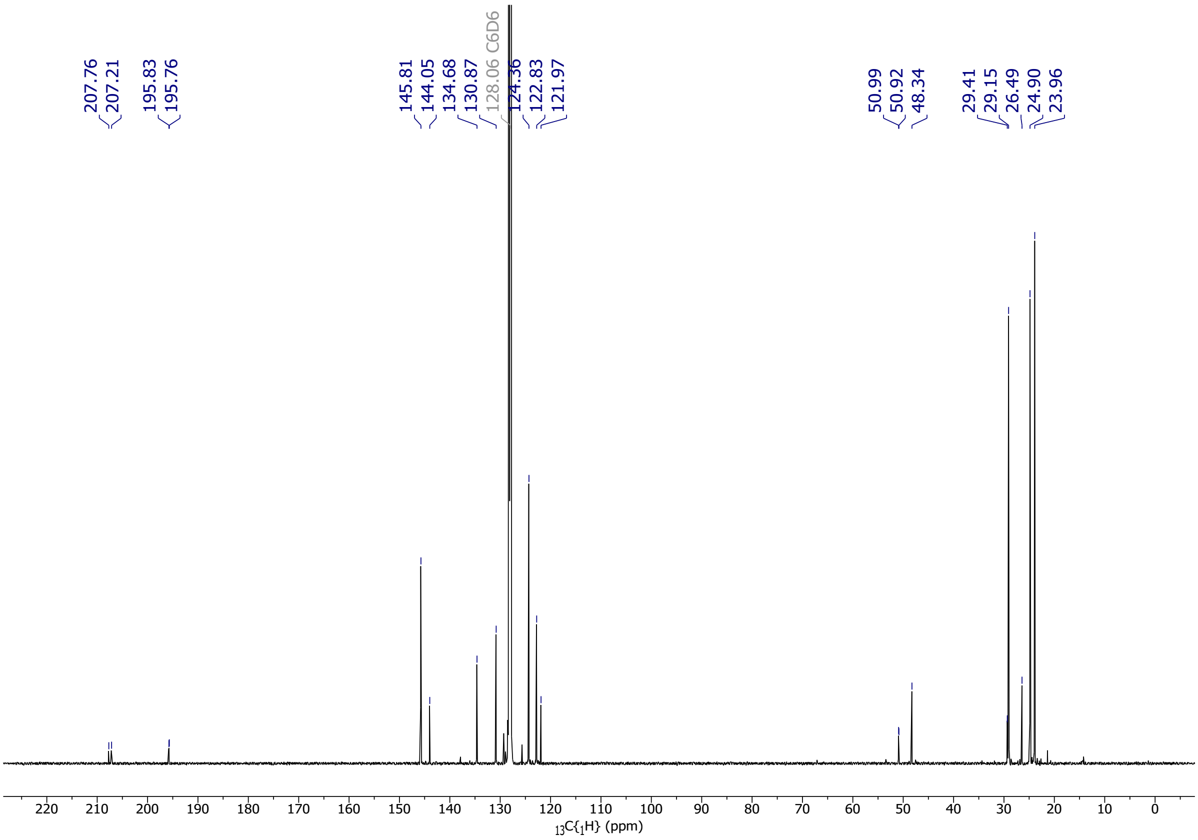

Figure S17. $^{13}C\{^{1}H\}$ NMR (151 MHz) spectrum of **4b** in C_6D_6.

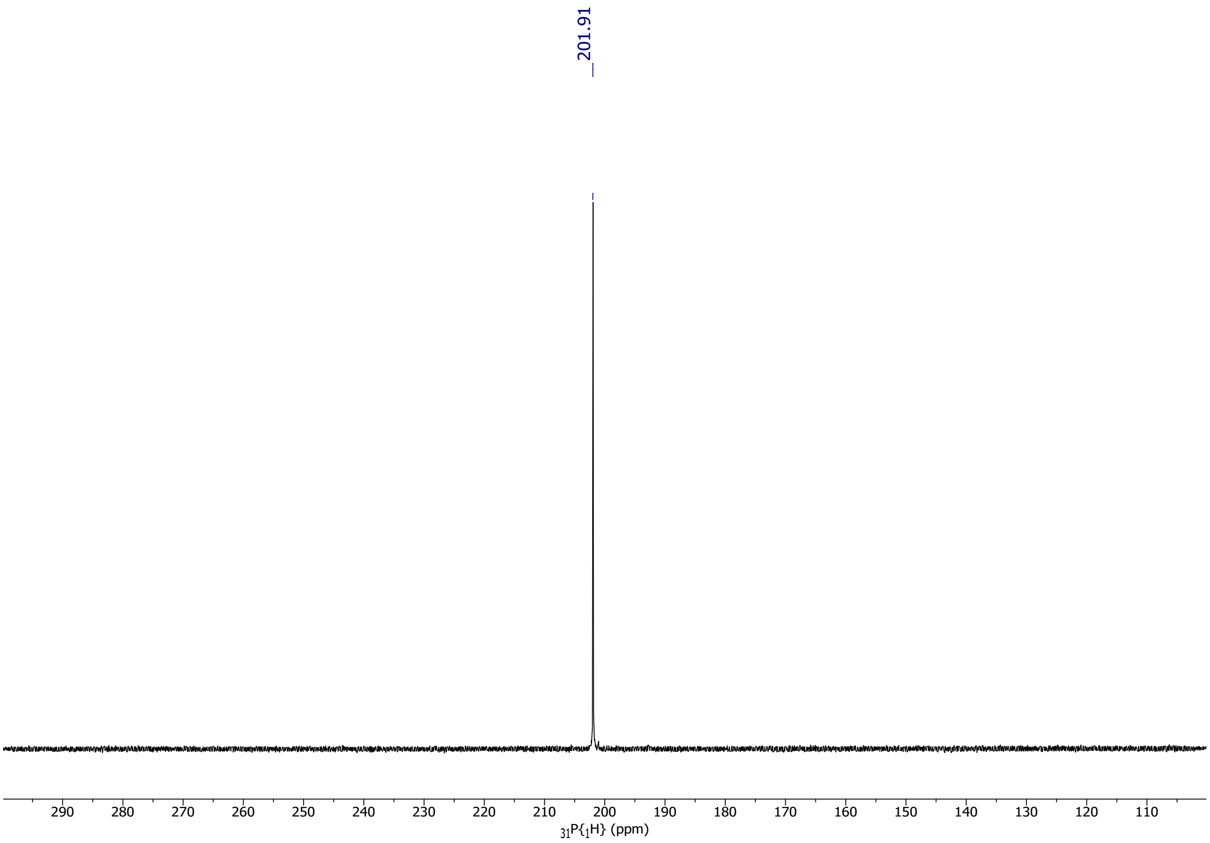

Figure S18. $^{31}P\{^{1}H\}$ NMR (243 MHz) spectrum of **4b** in C_6D_6.

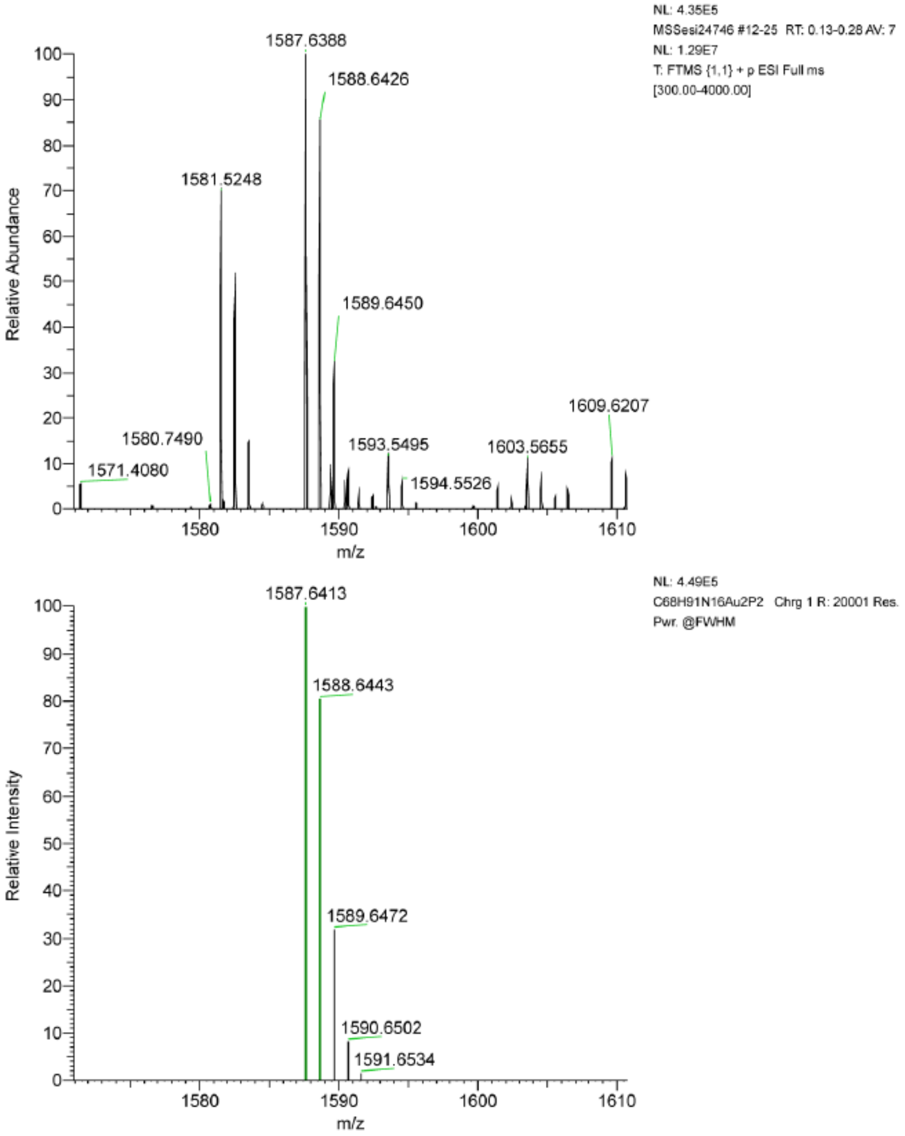

Figure S19. Top: high-resolution mass spectrum of **4b** showing the [M+H]$^+$ ion at *m*/*z* 1587.6388. Bottom: theoretical spectrum.

1.3 NMR Host-Guest binding studies

Host-guest binding constants for **P5A** and the axle precursors **1a** and **1b** were determined by ^{1}H NMR spectroscopic titration studies (500 MHz, C_6D_6, 298 K), wherein the perturbations in the **P5A** proton resonances 1 or 2 upon successive addition of **1a**/**1b** to a solution of **P5A** were fitted to a 1:1 host:guest binding stoichiometry using BindFit.[44] In both cases, calculated errors in the binding constant <10%.

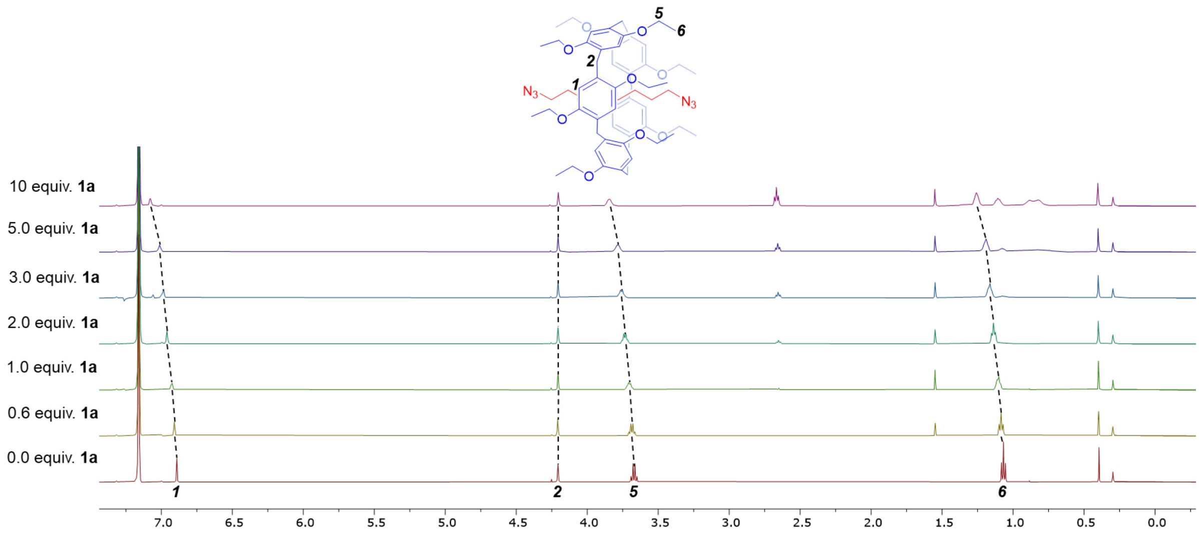

Figure S20. Stacked ^{1}H NMR spectra (500 MHz, C_6D_6, 298 K) of **P5A** upon successive addition of **1a**.

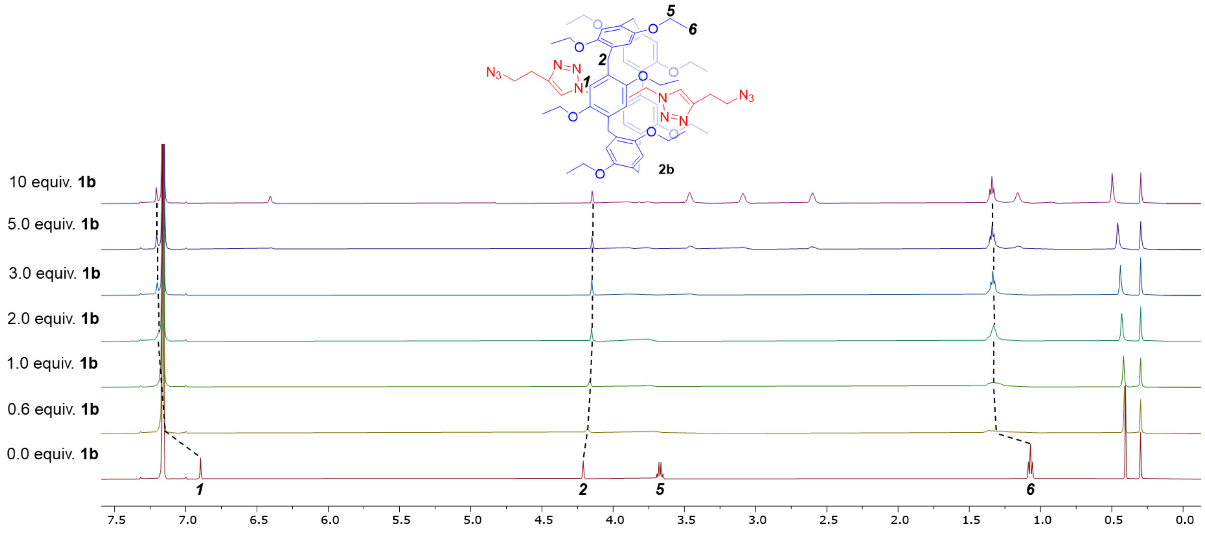

Figure S21. Stacked ^{1}H NMR spectra (500 MHz, C_6D_6, 298 K) of **P5A** upon successive addition of **1b**.

2. Single crystal X-ray diffraction data

2.1 X-ray data collection and refinement

Single-crystal X-ray diffraction data were collected using an Oxford Diffraction Supernova dual-source diffractometer equipped with a 135 mm Atlas CCD area detector. Crystals were selected under Paratone-N oil, mounted on micromount loops and quench-cooled using an Oxford Cryosystems open flow N_2 cooling device. Data were collected at 150 K using mirror monochromated Cu K_α (λ = 1.54184 Å) radiation and processed using the CrysAlisPro package, including unit cell parameter refinement and inter-frame scaling (which was carried out using SCALE3 ABSPACK within CrysAlisPro).[45] Structures were subsequently solved using direct methods.[46]

Table S1. Selected X-ray data collection/refinement parameters for **3a**·$3C_6H_6$ and **3b**.

	**3a**·$3C_6H_6$	**3b**
Formula	$C_{137}H_{176}Au_2N_{10}O_{10}P_2$	$C_{123}H_{164}Au_2N_{16}O_{10}P_2$
CCDC	2271341	2271342
Fw [g mol^{-1}]	2578.74	2482.57
Crystal system	triclinic	monoclinic
Space group	*P*1	$P2_1/n$
a (Å)	12.5314(2)	12.3413(2)
b (Å)	14.0421(2)	43.5798(6)
c (Å)	19.8345(3)	12.7293(2)
α (°)	96.403(1)	90
β (°)	107.769(1)	95.536(2)
γ (°)	95.770(1)	90
V (Å^3)	3269.42(9)	6814.29(18)
Z	1	2
Radiation, λ (Å)	Cu Kα, 1.54184	Cu Kα, 1.54184
Temp (K)	150(2)	150(2)
ρ_{calc} (g cm^{-3})	1.310	1.210
μ (mm^{-1})	4.856	4.653
Reflections collected	79947	107442
Indep. reflections	25632	11989
Parameters	1267	927
R(int)	0.0449	0.0937
R1/wR2,[a] I ≥ 2σI (%)	3.93/9.61	11.30/25.73
R1/wR2,[a] all data (%)	4.91/10.43	12.42/26.07
GOF	1.057	1.134

[a] $R1 = [\Sigma||F_o| - |F_c||]/\Sigma|F_o|$; $wR2 = \{[\Sigma w[(F_o)^2 - (F_c)^2]^2]/[\Sigma w(F_o^2)^2\}^{1/2}$; $w = [\sigma^2(F_o)^2 + (AP)^2 + BP]^{-1}$, where $P = [(F_o)^2 + 2(F_c)^2]/3$ and the A and B values are 0.0480 and 4.80 for **3a**·C_6H_6 and 0.040 and 128.18 for **3b**.

2.2 Details regarding the X-ray data collection and refinement for **3b**

Compound **3b** crystallizes as thin, weakly diffracting, colorless platelike crystals on slow diffusion of hexane into a concentrated solution of the sample in toluene. The selection of a suitable single crystal for XRD analysis was challenging due to the propensity of the platelike crystals to stack on top of one another. Despite numerous attempts at recrystallizing the sample from a range of different solvents, we were unable to obtain higher quality crystals. **3b** crystallizes in the centrosymmetric monoclinic space group $P2_1/n$ with the rotaxane located on a center of inversion. **3b** is a racemic mixture, with the two enantiomers being related by a center of inversion. Consequently, the asymmetric unit of **3b** contains half of the axle sub-unit, and half of the perethylated pillarene, **P5A**. The latter was modelled as one of the two enantiomers at 50% occupancy (with the other enantiomer being generated by a crystallographic symmetry element). While the data obtained are of insufficient quality to allow for the discussion of bond metric parameters, the single-crystal X-ray structure unequivocally confirms the formation of a mechanically interlocked rotaxane (see Figure S22), further supporting the conclusions drawn from NMR experiments and mass-spectrometry.

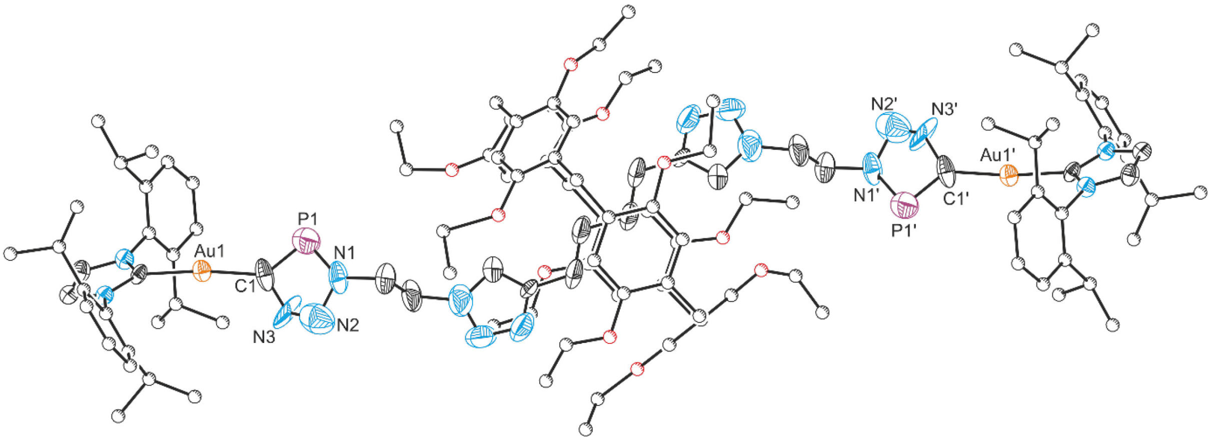

Figure S22. Single crystal XRD structure of **3b**. Anisotropic displacement ellipsoids depicted at 30% probability. Hydrogen atoms and solvent of crystallization omitted for clarity. Carbon atoms of Dipp substituents and of the **P5A** component pictured as spheres of arbitrary radius.

3. References

[44] D. Brynn Hibbert, P. Thordarson, *Chem. Commun.* **2016**, *52*, 12792–12805.

[45] *CrysAlisPro*, Agilent Technologies, Version 1.171.41.117a.

[46] (a) G. M. Sheldrick in *SHELXL97, Programs for Crystal Structure Analysis (Release 97-2)*, Institut für Anorganische Chemie der Universität, Tammanstrasse 4, D-3400 Göttingen, Germany, 1998; (b) G. M. Sheldrick, *Acta Crystallogr. Sect. A* **1990**, *46*, 467−473; (c) G. M. Sheldrick, *Acta Crystallogr. Sect. A* **2008**, *64*, 112−122.
